# A molecular basis behind heterophylly in an amphibious plant, *Ranunculus trichophyllus*

**DOI:** 10.1371/journal.pgen.1007208

**Published:** 2018-02-15

**Authors:** Juhyun Kim, Youngsung Joo, Jinseul Kyung, Myeongjune Jeon, Jong Yoon Park, Ho Gyun Lee, Doo Soo Chung, Eunju Lee, Ilha Lee

**Affiliations:** 1 School of Biological Sciences, Seoul National University, Seoul, Korea; 2 Department of Chemistry, Seoul National University, Seoul, Korea; 3 Plant Genomics and Breeding Institute, Seoul National University, Seoul, Korea; National University of Singapore and Temasek Life Sciences Laboratory, SINGAPORE

## Abstract

*Ranunculus trichophyllus* is an amphibious plant that produces thin and cylindrical leaves if grown under water but thick and broad leaves if grown on land. We found that such heterophylly is widely controlled by two plant hormones, abscisic acid (ABA) and ethylene, which control terrestrial and aquatic leaf development respectively. Aquatic leaves produced higher levels of ethylene but lower levels of ABA than terrestrial leaves. In aquatic leaves, their distinct traits with narrow shape, lack of stomata, and reduced vessel development were caused by *EIN3*-mediated overactivation of abaxial genes, *RtKANADIs*, and accompanying with reductions of *STOMAGEN* and *VASCULAR-RELATED NAC-DOMAIN7* (*VDN7*). In contrast, in terrestrial leaves, ABI3-mediated activation of the adaxial genes, *RtHD-ZIPIIIs*, and *STOMAGEN* and *VDN7* established leaf polarity, and stomata and vessel developments. Heterophylly of *R*.*trichophyllus* could be also induced by external cues such as cold and hypoxia, which is accompanied with the changes in the expression of leaf polarity genes similar to aquatic response. A closely-related land plant *R*. *sceleratus* did not show such heterophyllic responses, suggesting that the changes in the ABA/ethylene signaling and leaf polarity are one of key evolutionary steps for aquatic adaptation.

## Author summary

Evolutionary adaptation into aquatic environment is widely observed in diverse clades of land plants. To understand the molecular basis behind such adaptation, we analyzed *Ranunculus trichophyllus*, an amphibious plant producing different leaf shape depending on the growth conditions. Aquatic leaves of this plant produce higher levels of ethylene, which causes overactivation of genetic circuits composed of *EIN3*, an ethylene signaling transducer, and abaxial genes that suppress genes regulating stomata and xylem development. In contrast, terrestrial leaves produce higher levels of ABA, which activates adaxial genes and causes activation of stomata and xylem developments. Such changes in the ABA/ethylene signaling and leaf polarity after submergence were not observed in the closely-related land plant *R*. *sceleratus*, indicating that they are key evolutionary steps towards aquatic adaptation.

## Introduction

Since plants are sessile organisms, specific adaptations to their given environments are critical for their survival. Thus, compared to animals, plants show higher levels of phenotypic plasticity, differential phenotypic alterations exhibited in the same species depending on their surrounding environments [1, 2]. One of the most dramatic plant plasticity is heterophylly, an ability to produce morphologically different types of leaves depending on the growth environments [3]. Amphibious plants produce different shapes of leaves when grown under water compared to terrestrial growth; they usually produce thin and slender leaves in aquatic conditions but produce thick and stout leaves in terrestrial conditions [3]. Currently, the molecular mechanisms behind such heterophylly of amphibious plants are not well known.

Plant hormones participate in various plant developments so that plant architecture is shaped by the accurate regulation of the hormones [4, 5]. Plasticity by water adaptation is also regulated by plant hormones. Abscisic acid (ABA), auxin, ethylene, and gibberellin (GA) were proposed to mediate perception and responses to submergence into water [6, 7]. For example, auxin influences hyponastic growth and development of adventitious roots in submerged condition [6, 8]. Ethylene also regulates adventitious roots and rapid shoot growth when submerged, especially in deep water rice [7, 9]. Exogenous ABA treatment induces terrestrial leaf development in many aquatic plants whereas exogenous GA induces aquatic leaf development in some aquatic plants [3].

Leaf, as a photosynthetic organ, is a major plant organ showing plastic development depending on the environments [10, 11]. Leaves are developed from the shoot apical meristem as lateral organs and the leaf development is coordinated through three axes, a proximo-distal axis, an adaxial-abaxial (dorso-ventral) axis and medio-lateral axis. Adaxial-abaxial polarity has been well studied at the molecular level because establishment of this axis is critical for leaf morphogenesis [12]. Recent studies have identified several families of transcription factor genes determining adaxial and abaxial cell fate in the leaf [12, 13]. For example, *KANADI* (*KAN*) and *YABBY* (*YAB*) families and two *AUXIN RESPONSE FACTOR* genes (*ARF3/ETTIN* and *ARF4*) play critical roles in determination of abaxial cell fate whereas class III *homeodomain-leucine zipper* (*HD-ZIPIII*) genes, ARP (*ASYMMETRIC LEAVES1*, *ROUGH SHEATH2*, and *PHANTASTICA*) class Myb genes, and a LOB domain transcription factor, *ASYMMETRIC LEAVES2* (*AS2*), determine adaxial cell fate [14–18]. In addition, the expression domains of leaf polarity genes are finely delimited by small RNA; i.e., miR165/166 degrades *HD-ZIPIII* transcripts in abaxial side and tasiRNA erases the transcripts of *ARF3* and *ARF4* in adaxial side of the leaves [14, 19]. Likewise, abaxial fate-determining genes act antagonistically with adaxial fate-determining genes. For example, the *HD-ZIPIII* genes are ectopically expressed in abaxial side of the leaves in *kan1 kan2* double mutant, thus causing adaxialization [15]. In addition, overexpression of *KAN2* causes reduced expression of *PHB*, a *HD-ZIPIII* gene [15], indicating that *KAN* genes suppress the expression of *HD-ZIPIII*. In contrast, gain-of-function of *HD-ZIPIII* causes adaxialization whereas loss-of-function of *HD-ZIPIII* genes like *phb phv rev* triple mutant causes abaxialization, indicating that *HD-ZIPIII* genes antagonistically suppress *KANs* [13, 20]. Interestingly, both abaxialization and adaxialization cause partial radialization of the leaves.

Land plants have evolved from aquatic plants, algae, in Silurian period, ca. 400 million years ago [21]. Afterwards, they have developed various traits for land adaptation such as vascular structure, stomata and seed development [22, 23]. In addition, they have evolved a plant hormone, abscisic acid (ABA), and ABA signaling to endure dehydrated environments [24]. During the evolutionary process, diverse families of plants re-colonized water and turned into aquatic plants [25, 26]. Although derived from diverse clades of land plants, many submerged plants share common phenotypes such as thin and cylindrical leaves [3]. As submerged plants are subjected to the same selection pressure, there may be a common evolutionary developmental (evo-devo) mechanism modified from the genetic circuits present in the terrestrial plants. Such evo-devo mechanisms have yet to be disclosed.

In this study, we delved into the potential evo-devo adaptive mechanism of an amphibious plant, *Ranunculus trichophyllus* var. *kadzusensis*, which is an endangered species in Korea that lives in rice pad. We hypothesized that amphibious plants are an evolutionary bridge between land and aquatic plants, thus, the elucidation of adaptive molecular mechanism in *R*. *trichophyllus* would provide an insight how land plants re-adapted to aquatic environments. *Ranunculus* is a widespread genus containing hundreds of species adapted to various habitats in the northern hemisphere [27]. Many *Ranunculus* species live near the water, and some species adapted to aquatic environments [27, 28]. Therefore, *Ranunculus* genus is a good model system to investigate how land plants recolonized aquatic environments.

Here we show that the heterophylly of an amphibious plant, *R*. *trichophyllus*, is widely controlled by two plant hormones, ethylene and ABA. The protoplast transfection assays, at least in cellular level, demonstrated our hypothesis indicating that ethylene, increased at aquatic conditions, induces ETHYLENE INSENSITIVE3 (EIN3)-mediated overactivation of abaxial genes, *KANs*, and suppression of *STOMAGEN* (*STO*) and *VASCULAR-RELATED NAC-DOMAIN7* (*VDN7*), which cause cylindrical leaf morphology, lack of stomata, and reduced xylem development, three hallmarks of aquatic plants. In contrast, ABA, increased at terrestrial conditions, establishes leaf polarity through ABSCISIC ACID INSENSITIVE3 (ABI3)-mediated activation of adaxial genes, *HD-ZIPIIIs*, in adaxial side of the leaves, and induces *STO* and *VDN7* for the development of stomata and vessel elements. In addition, we show that molecular changes have occurred in the expressions of ABA biosynthetic gene and leaf polarity genes in aquatic *R*. *trichophyllus* compared to a land plant relative, *R*. *sceleratus*.

## Results

### Morphological traits showing heterophylly of *R*. *trichophyllus*

**Fig 1:**
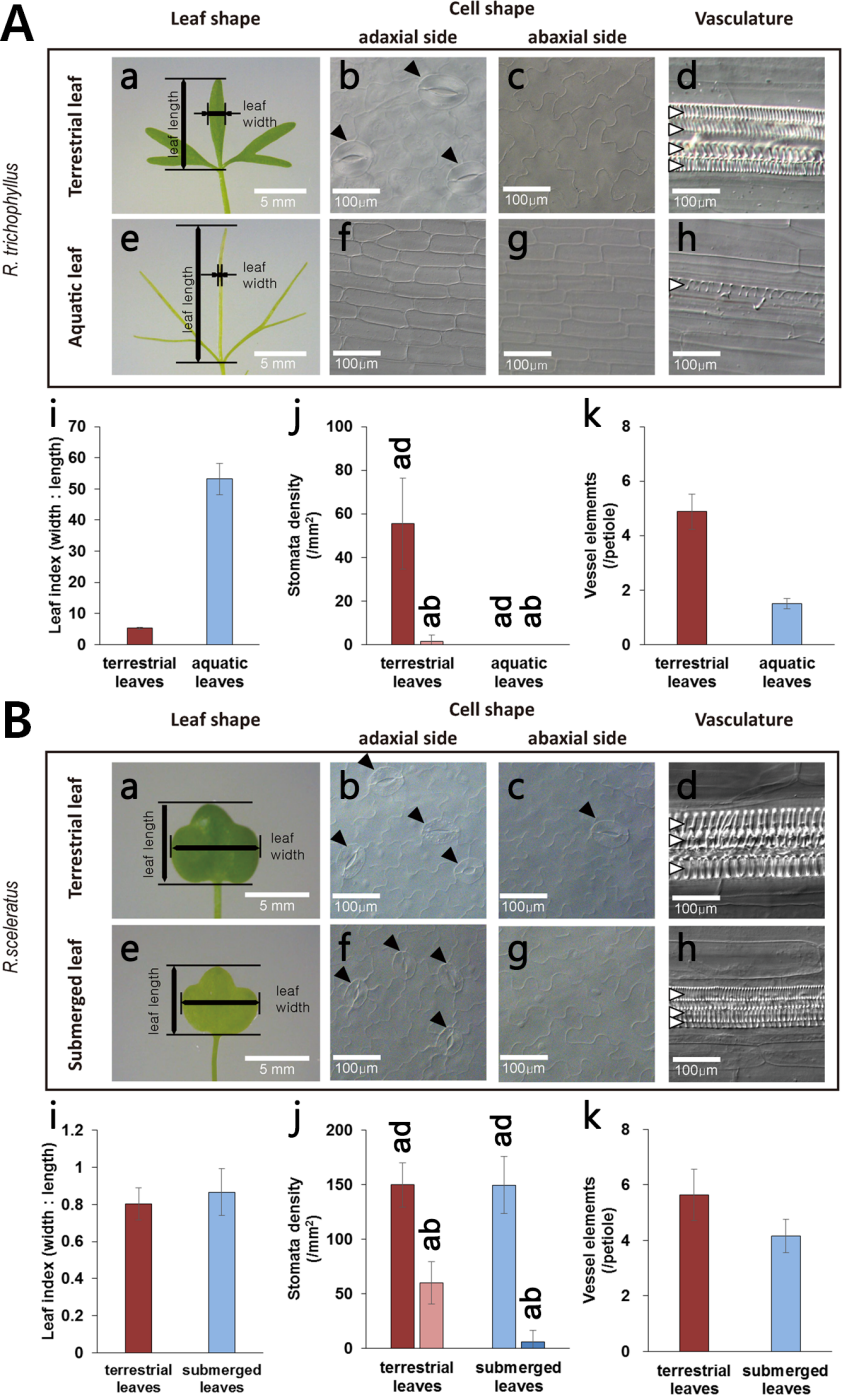
Heterophyllic leaf developments depending on environments are shown in *R*. *trichophyllus* but not in sister species, *R*. *sceleratus*. Seedling morphologies and microscopic images of *R*. *trichophyllus* (A) and *R*. *sceleratus* (B) grown under aerial vs aquatic environments. Seeds of *R*. *trichophyllus* or *R*. *sceleratus* were germinated on solid MS media for 1 week, then transferred to aerial or aquatic environments. The true leaves produced at 7 days after transference were used for analysis. (*a-d*) terrestrial and (*e-h*) aquatic/submerged plants, (*b*, *c*) cell shapes of terrestrial leaves and (*f*, *g*) those of aquatic/submerged leaves, (*d*) vasculature of terrestrial and (*h*) that of aquatic leaves. (*i-k*) Statistical analyses of leaf indices (*i*), stomatal densities (*j*), and number of vessel elements (*k*) in terrestrial and aquatic/submerged leaves. Data are collected from 24 individual samples and presented as means ± SD from three biological replicates. Black arrowheads denote stomata and white arrowheads denote individual vessel element.

*Ranunculus trichophyllus* is an amphibious plant that grows both on land and under water. Depending on their growth environments, they develop morphologically different types of leaves (Fig 1). Under terrestrial conditions, *R*. *trichophyllus* develops thick and broad leaves whereas under aquatic conditions it produces thin and cylindrical leaves (Fig 1A). The leaf index, a leaf length-to-width ratio, in aquatic leaves was approximately 10-fold higher than that in terrestrial leaves (Fig 1Ai), which is an indicative of slender appearance of aquatic leaves. Microscopic analyses showed that terrestrial leaves have well-developed stomata, particularly on adaxial surfaces, whereas aquatic leaves completely lack stomata (Fig 1Ab, c, f and g). When comparing cell structure, terrestrial leaves showed stout and irregular-shaped epidermal cells, whereas aquatic leaves showed slender and rectangular epidermal cells. We also observed that terrestrial leaves have a higher number of developed vessel elements than aquatic leaves (Fig 1Ad and h). In contrast, a close sister species *R*. *sceleratus*, which lives near the waterside, does not show such heterophylly (Fig 1B). The leaf index and leaf morphology of *R*. *sceleratus*, were not affected by 1 week submergence. In addition, stomata density and the number of vessel elements were little changed by submergence (Fig 1B). Moreover, *R*. *sceleratus* showed severe growth retardation by 3 weeks of long-term submergence, which is similar to *Arabidopsis thaliana* (S1 Fig). It suggests that the heterophyllic response of *R*. *trichophyllus* is evolutionarily adaptive trait for long-term submergence.

### Transcriptomic analysis of *R*. *trichophyllus*

**Fig 2:**
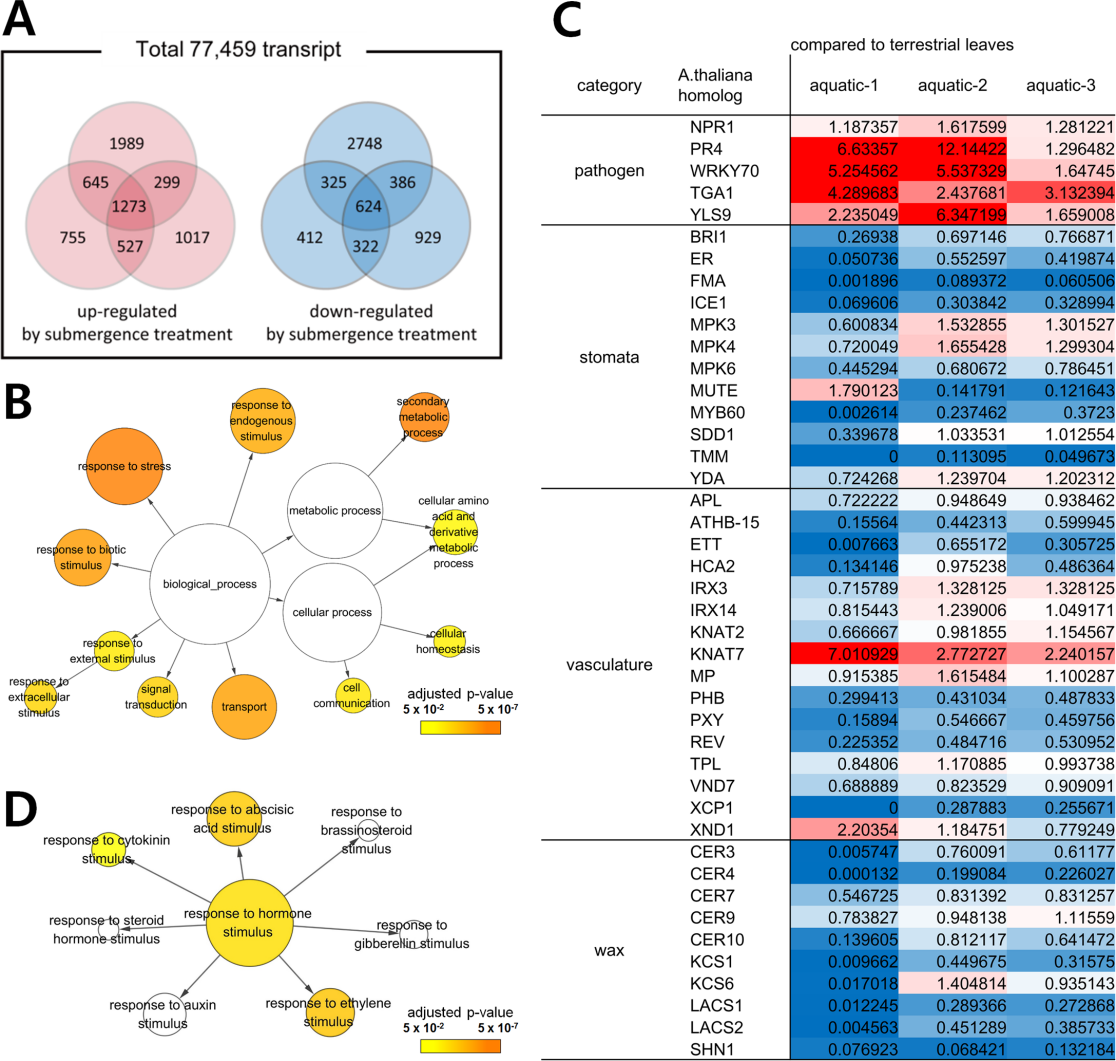
Transcriptome analysis of aquatic vs terrestrial plants of *R*. *trichophyllus*. (A) Venn diagram of differentially expressed transcripts with two fold changes for three independent experiments. Numbers are up- or down-regulated genes in aquatic plants compared to terrestrial plants. (B) Diagram for large ontology categories showing up-regulation in aquatic plants by BinGo software. Number of genes is represented by relative size of circles that belong to each gene ontology term. (C) Relative expression of genes affiliated to four developmental GO terms for terrestrial vs aquatic plants of *R*. *trichophyllus*. Up-regulated genes are painted with red and down-regulated genes are painted with blue. (D) Diagram of up-regulated genes in aquatic plants for ontology categories of plant hormone response genes by the BinGo software. The seedlings, 1 week-old after germination, were transferred to terrestrial or aquatic condition for 10 days. Upper parts of seedlings including leaves and shoot apexes were harvested for RNA sequencing.

To understand the molecular basis of heterophylly in *R*. *trichophyllus*, we performed quantitative whole gene expression analysis using terrestrial leaves vs aquatic leaves by RNA sequencing. A total of 77,459 transcripts were analyzed, and ca. 15.8% of transcripts were up- or down-regulated in aquatic leaves compared to terrestrial leaves (Fig 2A). In general, the genes involved in the response to internal and external stimuli and stress-response genes showed significant up-regulation in aquatic plants (Fig 2B). Among the Gene Ontology (GO) terms related to stress response, ‘response to hypoxia’ (GO:0001666), ‘defense response’ (GO:0006952), ‘response to osmotic stress’ (GO:0006970) were prominent for up-regulation in aquatic plants, which may have evolved to protect plants from environmental stimuli and hypoxia stress in aquatic environments. We also found that genes involved in stomata and vascular developments were considerably down-regulated in aquatic leaves, which reflect lack of stomata and underdeveloped vessel elements. Moreover, well-known pathogen-resistance genes were up-regulated and genes for wax biosynthesis were down-regulated (Fig 2C). More importantly, the transcriptome analysis clearly pointed out that the two plant hormones, ethylene and ABA, are related to heterophyllic leaf development (Fig 2D). Such transcriptional changes may be required for evolutionary adaptation into aquatic environments. To test this hypothesis, we analyzed the effects of submergence on the expressions of the orthologous genes from *R*. *sceleratus*. In contrast to *R*. *trichophyllus*, the orthologous genes from *R*. *sceleratus* showed no significant differential expression in response to submergence (S2 Fig).

### Hormonal regulation of heterophyllic development

**Fig 3:**
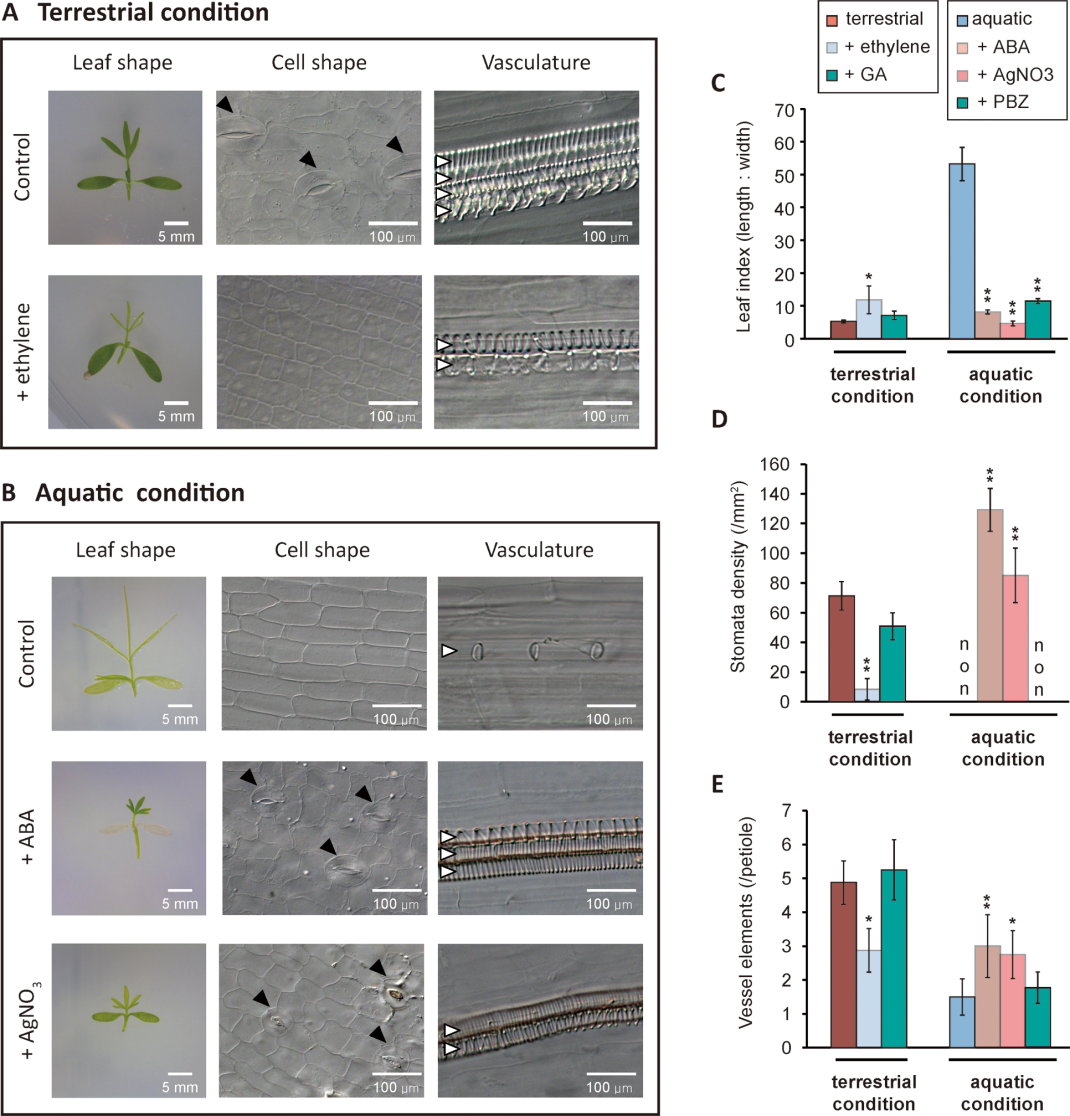
Ethylene and ABA control heterophylly of *R*. *trichophyllus*. (A) Images of seedlings, stomata, and vessel elements for terrestrial leaf and the leaf after ethylene treatment. (B) Images of seedling, stomata, and vessel elements for aquatic leaf and the leaf after treatment of ABA and AgNO_3_, an ethylene inhibitor. (C-E) Statistical analyses of leaf indices (C), stomatal densities (D), and number of vessel elements (E) after treatment with hormones (ethylene, ABA, and GA) and hormone inhibitors (AgNO_3_ and PBZ). Data are collected from 16–24 individual samples and presented as means ± SD from three biological replicates. Black arrowheads denote stomata and white arrowheads denote individual vessel element. *P < 0.05; **P < 0.01 (unpaired Student’s *t*-test).

In addition to our transcriptome analysis, there are studies showing that ethylene, and GA can cause land-grown amphibious plants to develop an aquatic leaf-like morphologies [29, 30]. Thus, we wondered if any of the plant hormones effect on the heterophyllic development of *R*. *trichophyllus* seedlings (Fig 3). We found that exogenous ethylene treatment of terrestrial plants caused an increase of the leaf index, reduced number of stomata and vessel elements, whereas treatment of the aquatic leaves with silver nitrate (AgNO_3_), an inhibitor of ethylene biosynthesis, caused the opposite effects such that decreased leaf index and increased the number of stomata and vessel elements (Fig 3A). In contrast, when aquatic plants were treated with ABA, the leaf index was dramatically reduced whereas the numbers of stomata and vessel elements were increased (Fig 3B). GA treatment on the terrestrial plants did not reduce the number of stomata and vessel elements (Fig 3D and 3E). Likewise, paclobutrazol (PBZ), an inhibitor of GA biosynthesis, did not affect to stomata and vasculature development even though the leaf index was decreased (Fig 3C–3E). In addition, auxin and brassinosteroid (BR) treatments caused almost no effect (S3 Fig). The results suggest that aquatic leaf morphologies of *R. trichophyllus* are dependent on ethylene whereas terrestrial ones are dependent on ABA. GA, auxin, and BR do not appear to be involved in the heterophylly of *R*. *trichophyllus*. In contrast to *R*. *trichophyllus*, *R*. *sceleratus* did not show any morphological changes in response to ethylene and ABA (S4 Fig), indicating that ethylene and ABA signaling could control leaf development in *R*. *trichophyllus* but not in *R*. *sceleratus*.

### ABA and ethylene mediate heterophyllic development

**Fig 4:**
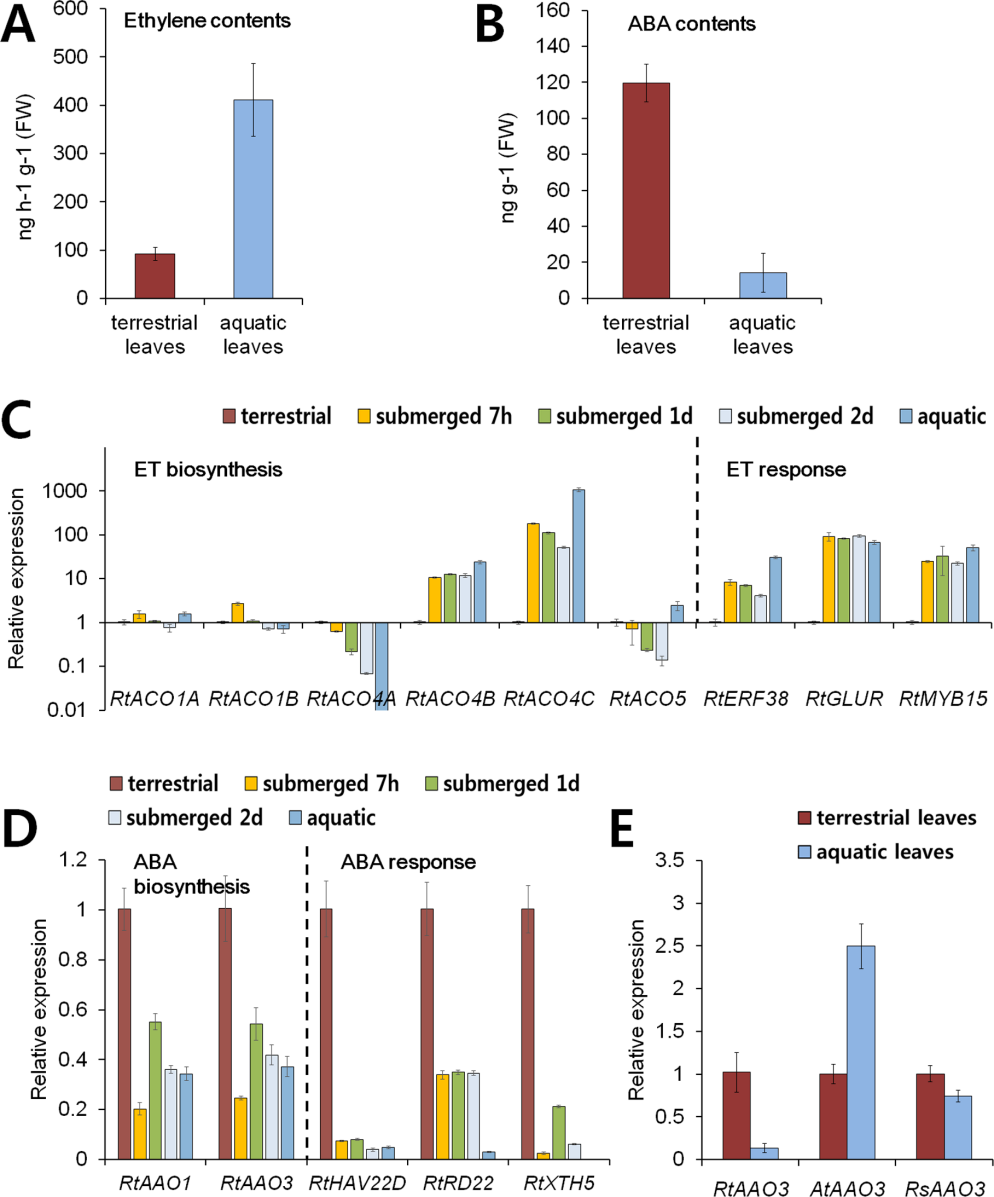
Differential expression of ethylene and ABA-related genes under terrestrial and aquatic environments is specific to amphibious *R*. *trichophyllus*. (A and B). Contents of ethylene (A) and ABA (B) in terrestrial vs aquatic leaves. (C and D). Comparison of transcript levels of ethylene- (A) and ABA- (B) biosynthesis and responsive genes following submergence into water. Plants harvested at 7 hours (7h), 1 day (1d), and 2 days (2d) after submergence were compared with terrestrial and aquatic plants for expressions. (E) Effects of submergence on the expression levels of *AAO3* genes in *R*. *trichophyllus*, Arabidopsis, and *R*. *sceleratus*. For submergence, two weeks old plants grown on solid MS media were submerged into water for 5 days for RNA extraction. The data represent means ± standard error from three biological and two technical replicates.

Since the treatments of plant hormones indicated that ABA and ethylene mediates heterophyllic leaf development of *R*. *tricophyllus*, we analyzed the contents of ABA and ethylene in terrestrial and aquatic leaves (Fig 4A and 4B). As expected, terrestrial leaves contained 3 times higher level of ABA than aquatic leaves whilst aquatic leaves contained 4 times higher level of ethylene than terrestrial leaves. Then, we checked if expressions of any specific genes encoding the enzymes involved in the critical steps of ABA and ethylene biosynthetic pathways are differentially regulated according to environments. The gene encoding enzyme for critical step of ABA biosynthesis is *ABA Aldehyde Oxidase* (*AAO*) and that for ethylene biosynthesis is *ACC Oxidase* (*ACO*) [31, 32]. Thus, we cloned the orthologs of *AAO* and *ACO* from *R. trichophyllus* and compared the expression levels depending on the growth condition (Fig 4C and 4D). Consistent with the hormonal contents, terrestrial plants showed higher expression of *RtAAO* than aquatic plants whereas aquatic plants showed higher expression of *RtACO* than terrestrial plants in general. For ethylene biosynthesis, *RtACO4B* and *RtACO4C* showed remarkable increase in aquatic plants compared to terrestrial plants (Fig 4C). To address if submergence of land plants into water causes rapid changes in the expression of ABA/ethylene biosynthesis genes, we checked dynamic expressions of the genes at 7 hours, 1 day, and 2 days after submergence. The results showed that ethylene-biosynthesis genes, *RtACO4B* and *RtACO4C*, showed rapid increase within 7 hours, then slow increase until 2 days after submergence (Fig 4C). In case of ABA-biosynthesis genes, *RtAAO1* and *RtAAO3*, the transcript levels were highly decreased within 7 hours of submergence but slowly increased afterwards (Fig 4D). We also checked if genes responsive to ABA and ethylene are increased in terrestrial and aquatic leaves of *R*. *trichophyllus* respectively. As expected, aquatic leaves showed higher expression of ethylene-responsive genes whereas terrestrial leaves showed higher expression of ABA-responsive genes (Fig 4C and 4D). Moreover, dynamic expression patterns of ABA/ethylene responsive genes after submergence showed rapid changes within 7 hours after submergence. Such results suggest that aquatic condition triggers *in vivo* ethylene signaling cascades and suppresses ABA signaling pathway.

In addition, we found that although submergence of *R*. *trichophyllus* into water rapidly downregulates expression of the ABA biosynthesis gene, *RtAAO3* (*ABA-aldehyde oxidase*), expression of *AtAAO3*, an Arabidopsis ortholog, is not reduced, instead increased by submergence, perhaps due to hypoxic stress (Fig 4E). In *R*. *sceleratus*, a waterside plant, the expression of ortholog, *RsAAO3*, was reduced relatively weakly by submergence (Fig 4E). This result suggests that the suppression of ABA biosynthesis in aquatic environments is an evolutionary adaptation developed in amphibious *R*. *trichophyllus*.

### Differential expression of leaf polarity genes according to growth environments

**Fig 5:**
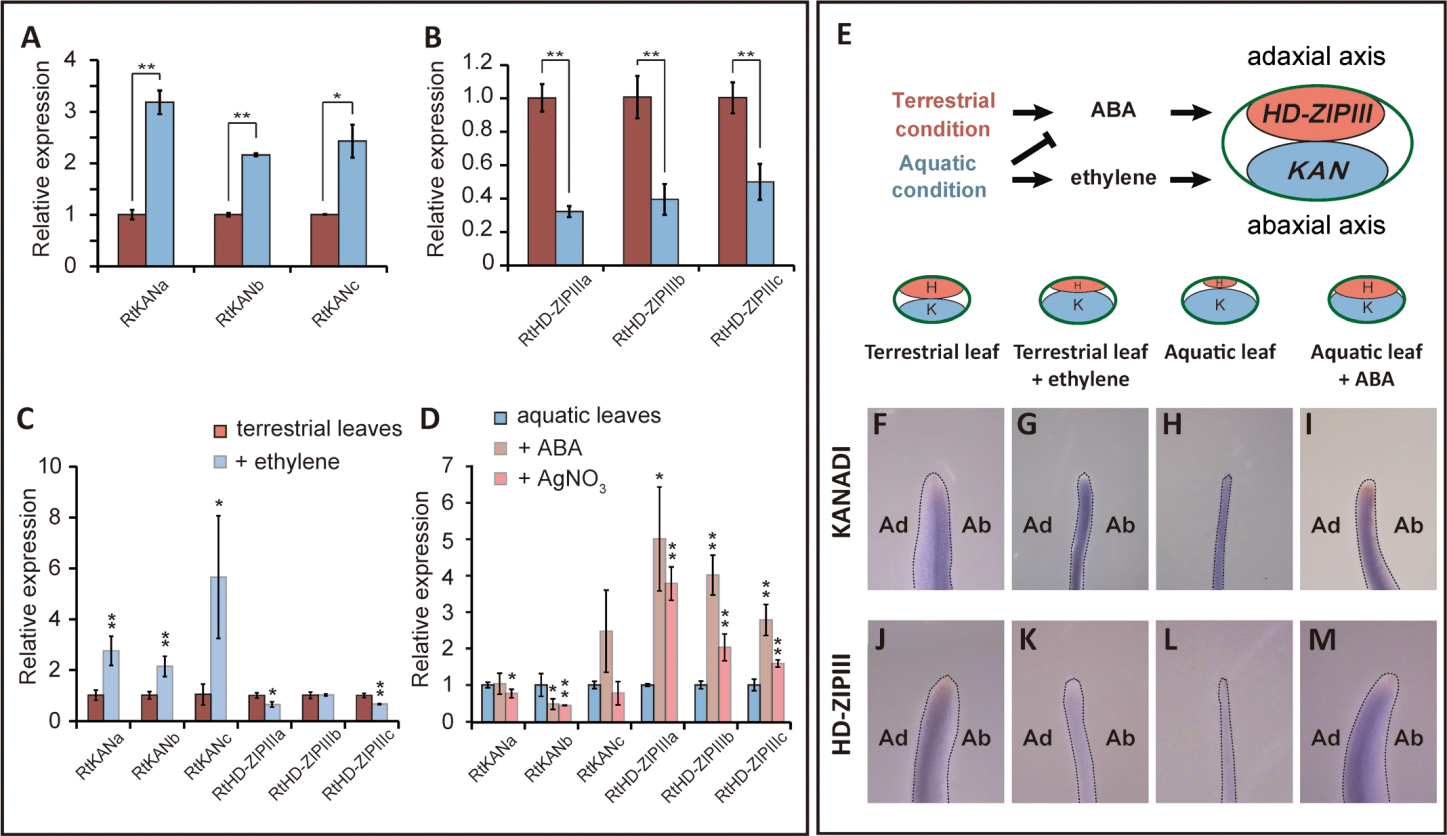
Expressions of leaf polarity genes, *KANs* and *HD-ZIPIIIs* are dependent on the environment. (A and B) Gene expression analyses of *KAN* genes (A), and *HD-ZIPIII* genes in terrestrial and aquatic leaves. (C and D) Transcript levels of leaf polarity genes after chemical treatments. The data are presented as means ± SD from three biological and two technical replicates. ACC, an ethylene precursor, was treated as ethylene. *P < 0.05; **P < 0.01 (unpaired Student’s *t*-test) (E) Model of heterophyllic developments regulated by ABA and ET activating leaf polarity genes, *KANs* and *HD-ZIPIIIs*, respectively. K; *KANADIs*, H; *HD-ZIPIIIs*. (F-M) Whole mount *in situ* hybridization for *RtKANa* (F-I) and *RtHD-ZIPIIIa* (J-M). Ab, abaxial side; Ad, adaxial side.

To address the molecular mechanism behind heterophyllic leaf development, we explored the roles of several leaf development genes in the differential leaf morphologies in aquatic and terrestrial environments. Since the leaf structure and morphology is mainly governed by leaf polarity genes, we cloned three *KAN* and three *HD-ZIPIII* homologs, which determine abaxial and adaxial identity, respectively [14–16]. We named these genes *KANa*, -*b*, and -*c*, and *HD-ZIPIIIa*, -*b*, and -*c* (S5 Fig). Overexpression of *RtKANa* and *RtHD-ZIPIIIa* in Arabidopsis caused narrow or curling leaf morphology, which phenocopied the transgenic lines overexpressing Arabidopsis homologs (S5C Fig) [14, 16, 33]. The expression of the abaxial genes, *RtKANs*, was much higher in aquatic than in terrestrial leaves, suggesting that *RtKANs* are overexpressed in aquatic environments. In contrast, expression of adaxial genes, *RtHD-ZIPIIIs*, was significantly reduced in aquatic leaves (Fig 5A and 5B). *In situ* hybridization showed that *RtKANa* expression is mainly detectable around the midvein and abaxial side, but is not detectable in the adaxial side of terrestrial leaves (Fig 5F and S6 Fig). However, strong expression of *RtKANa* throughout aquatic leaves was observed (Fig 5H). In contrast, the expression domain of *RtHD-ZIPIIIa* was confined to the adaxial side of terrestrial leaves and was barely detectable in aquatic leaves (Fig 5J and 5L). These findings strongly support the hypothesis that the axial expressions of *RtKANs* and *RtHD-ZIPIIIs* are perturbed in aquatic leaves. In contrast to *R*. *trichophyllus*, Arabidopsis and *R*. *sceleratus* did not show any such alteration of the polarity gene expression following submergence (S7 Fig), indicating that these traits are acquired during re-adaptation to water.

Next, we investigated whether the expressions of leaf polarity genes are affected by ethylene and ABA. Under the terrestrial condition, treatment of plants with ethylene resulted in increased expression of the abaxial genes, *RtKANs*, thus, phenocopying aquatic leaves. However, the expression of adaxial genes, *RtHD-ZIPIIIs*, were not significantly affected (Fig 5C). Under aquatic condition, treatment of plants with ABA and AgNO_3_ led to increased expression of *RtHD-ZIPIII* genes, although no remarkable decrease in *RtKAN* expression was observed (Fig 5D). These results support the hypothesis that ethylene activates *RtKANs*, whereas ABA activates *RtHD-ZIPIIIs* as shown in the model (Fig 5E). The RNA expression pattern observed by *in situ* hybridization also supported these results: ethylene treatment increased the expression domain of *RtKAN* in terrestrial leaves whereas ABA treatment increased that of *RtHD-ZIPIIIa* in aquatic leaves (Fig 5I and 5M).

### Hormonal signaling cascades to leaf polarity genes

**Fig 6:**
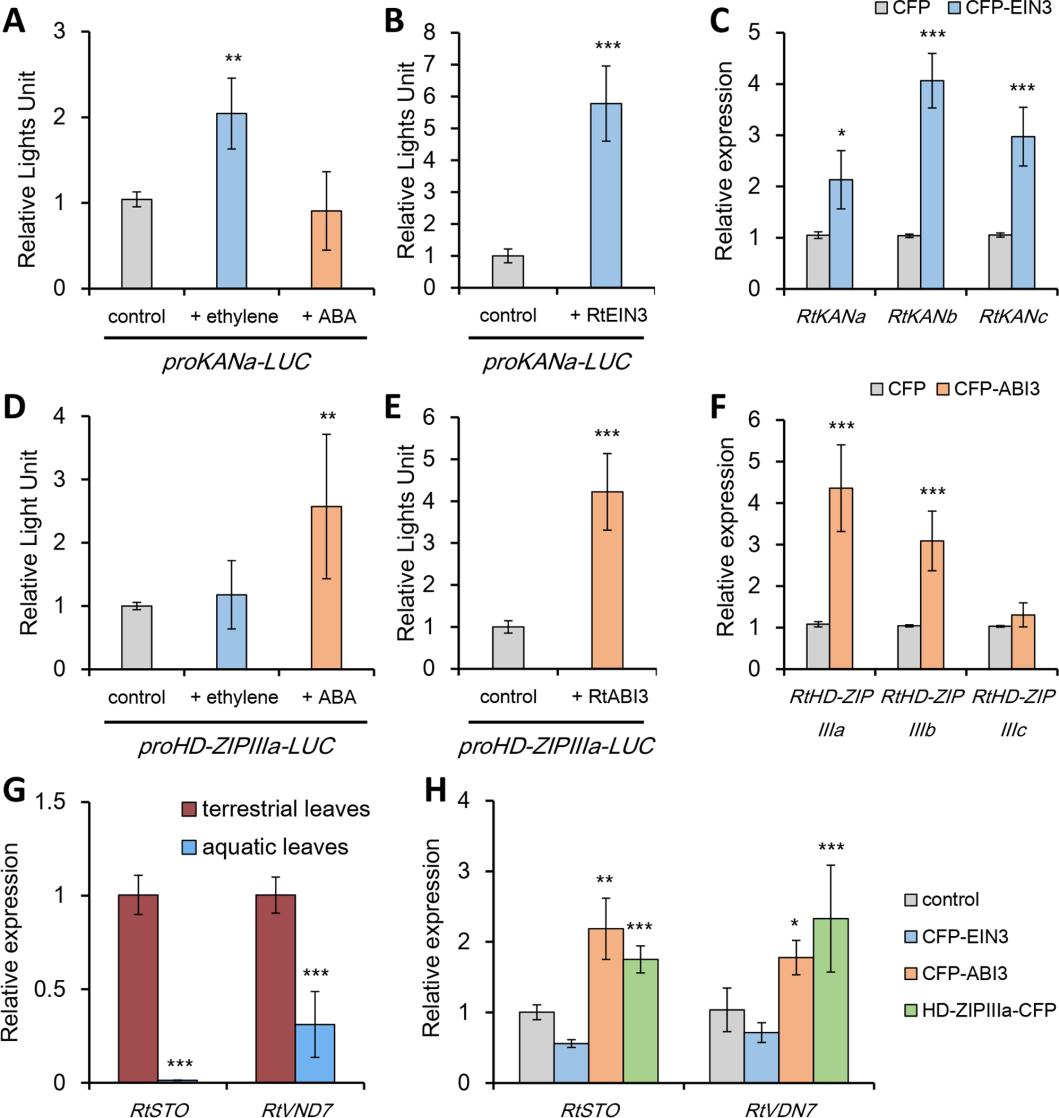
Effects of ethylene and ABA signalings on the promoter activation of leaf polarity genes and genes critical for stomata and vascular developments. (A and B) Luminescence analysis of *proRtKANa*::*LUC* when added with ACC, an ethylene precursor, or ABA in protoplast solution (A), control; without chemical treatment, and when cotransfected with *CFP-RtEIN3* in protoplasts (B), control; cotransfected with *CFP* construct. (C) Effects of *CFP-RtEIN3* transfection on the relative expressions of endogenous *KAN* genes in protoplasts, control; transfected with *CFP* construct. (D and E) Luminescence analysis of *proHD-ZIPIIIa*::*LUC* when added with ACC or ABA in protoplast solution (D), control; without chemical treatment, and when cotransfected with *CFP-RtABI3* in protoplasts (E), control; cotransfected with CFP construct. (F) Effects of *CFP-RtABI3* transfection on the relative expressions of endogenous *HD-ZIPIII* genes in protoplasts, control; transfected with *CFP* construct. (G) Relative transcript levels of *RtSTO* and *RtVDN7*, encoding critical regulators of stomata and vascular developments, when transfected with *Rt-EIN3*, *Rt-ABI3*, and *RtHD-ZIPIIIa* fused with *CFP* coding sequence. Control; transfected with *CFP* construct. (H) Comparison of transcript levels of *RtSTO* and *RtVND7* between terrestrial and aquatic leaves of *R*. *trichophyllus*. *P < 0.05; **P < 0.01; ***P < 0.001 (unpaired Student’s *t*-test).

To address if ABA and ethylene directly regulate leaf polarity genes, *RtHD-ZIPIIIs* and *RtKANs*, we developed a *Ranunculus* protoplast transient expression assay using seedlings grown on solid MS media. The promoters of *RtKANa* and *RtHD-ZIPIIIa* were fused to the luciferase reporter gene (*LUC*) and tested for their response to ethylene and ABA in transiently transfected protoplasts. As expected, *RtKANa* promoter was rapidly induced by ethylene but was not affected much by ABA whereas the *RtHD-ZIPIIIa* promoter was strongly induced by ABA but was not significantly affected by ethylene (Fig 6A and 6D). This indicates that ethylene and ABA directly regulate the promoters of *RtKANa* and *RtHD-ZIPIIIa* respectively. Then, we searched for candidate transcription factors that might mediate the ethylene and ABA signaling by directly acting on the promoters of *RtKANs* and *RtHD-ZIPIIIs*. Interestingly, when *RtEIN3* is cotransfected with *proKANa-LUC* into protoplasts, it caused strong activation of luciferase activity. Similarly, when *RtABI3* is cotransfected with *proHD-ZIPIIIa-LUC*, it caused strong activation of luciferase activity (Fig 6B and 6E). This indicates that RtEIN3 and RtABI3 directly activate *RtKANs* and *RtHD-ZIPIIIs* respectively. Consistently, transfected *RtEIN3* increased expressions of all three endogenous *RtKAN* genes and transfected *RtABI3* increased all of *RtHD-ZIPIIIs* (Fig 6C and 6F), which supports the model shown in Fig 4E.

Finally, we wondered if ethylene and ABA signalings directly control heterophyllic leaf development through transcriptional cascades. Thus, we transfected upstream transcription factors, *EIN3, ABI3*, and *HD-ZIPIII* into protoplasts, then checked the expression of two key regulators controlling stomata and vessel developments; *STO*, encoding a peptide protein turning on stomatal development[34], and *VDN7*, encoding a NAC domain transcription factor controlling vascular development.[35] *RtSTO* and *RtVND7* were down-regulated in aquatic leaves, which are consistent with the lack of stomata and reduced number of vessel elements in aquatic leaves (Fig 6G). Protoplast transfection assays showed that transient overexpression of *RtABI3* or *RtHD-ZIPIIIa* increases transcript levels of *RtSTO* and *RtVDN7*, suggesting that stomata and vessel developments in terrestrial leaves are controlled by an ABI3-RtHD-ZIPIIIa regulatory module (Fig 6H). In contrast, overexpression of *RtEIN3* decreases transcript levels of *RtSTO* and *RtVDN7*. Taken together, ethylene and ABA signaling control leaf polarity, stomata development and vascular development, the three hallmarks of heterophyllic development in *R*. *trichophyllus*.

### Cold and hypoxia induce aquatic leaf development

**Fig 7:**
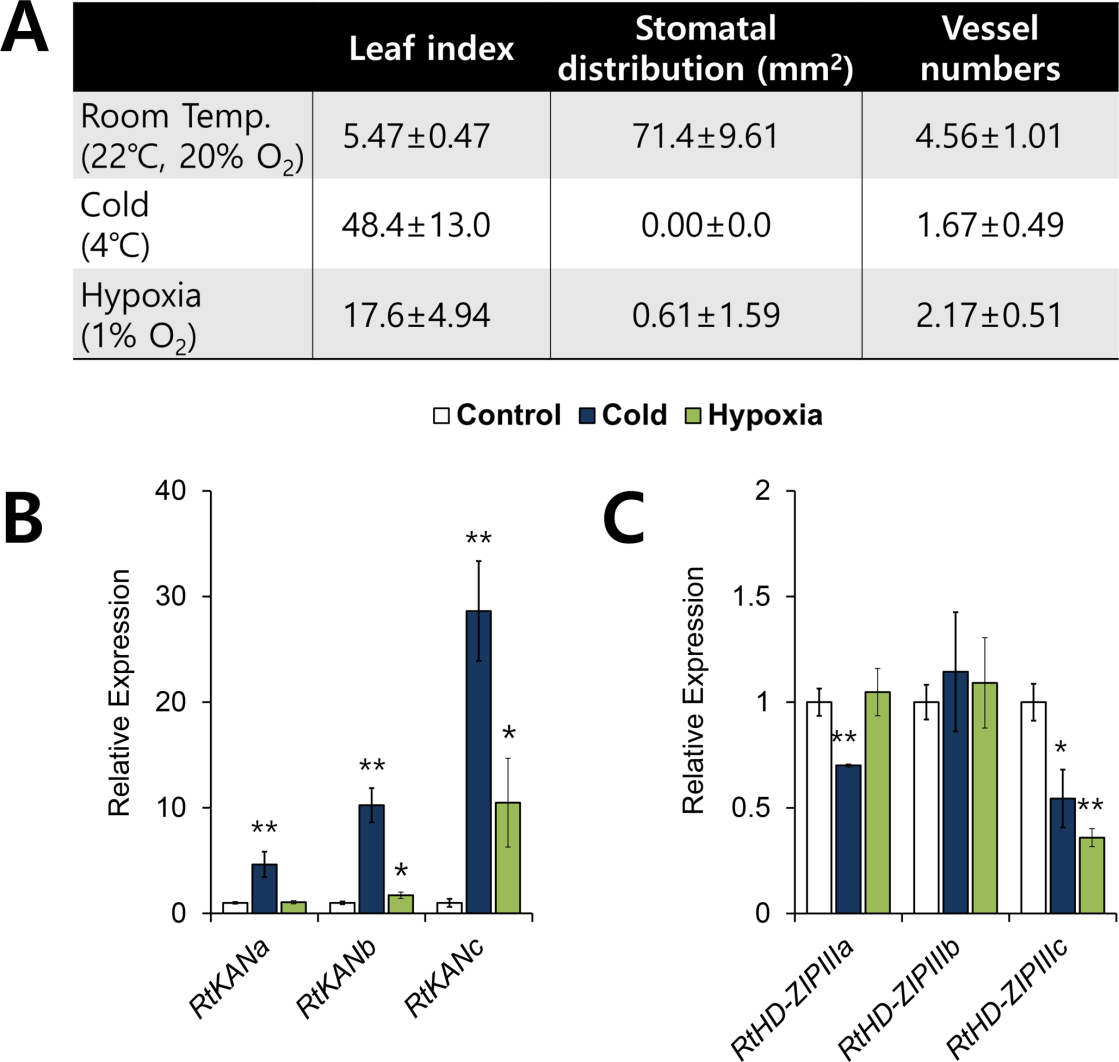
Cold and hypoxia induce aquatic leaf development in *R*. *trichophyllus*. (A) Heterophylly induced by cold and hypoxia. 1 week-old seedlings after germination on the MS media were transferred to cold chamber (4°C) for 1 month or hypoxia chamber (1% O_2_) for 2 weeks. The column Room Temp is a control at 22°C with 20% O_2_. (B and C) Gene expression analyses of *KAN* genes (B), and *HD-ZIPIII* genes (C) in the leaves after cold for 1 month and hypoxia for 2 weeks treated. *P < 0.05; **P < 0.01 (unpaired Student’s *t*-test).

It has been reported in some species of amphibious plants that certain environmental conditions such as cold cause aquatic leaf development mimicking aquatic condition [36]. Thus, we checked if any environmental conditions cause aquatic leaf development in *R*. *trichophyllus* (Fig 7). We found that 4°C cold temperature and hypoxia (less than 1% O_2_ concentration) caused significant increase of leaf index. In addition, the plants grown at cold temperature showed lack of stomata and decrease of vessel numbers, indicating that cold temperature mimics the aquatic condition well. However, hypoxia caused reduced number of stomata and vessel elements, suggesting that hypoxia mimics aquatic condition partially (Fig 7A). Then, we checked if cold and hypoxia effect on the expressions of leaf polarity genes similar to aquatic condition (Fig 7B and 7C). As expected, expression of *KAN* genes was higher whereas that of *HD-ZIPIII* genes was lower in the plants grown under cold temperature compared to room temperature. Consistent with the phenotypic effect, hypoxia caused less effects on the expression of both polarity genes than cold temperature. This result suggests that environmental cues inducing aquatic leaf development also cause similar molecular changes in *R*. *trichophyllus*.

## Discussion

Although embryophytes, land plants, have evolved from water to land by acquiring land adaptation such as vascular development and broad-leaf morphology, diverse plant species from different phylogenetic clades have returned to aquatic environments, indicating that water re-adaptation is quite common [21, 37]. *R*. *trichophyllus* is an amphibious plant, an evolutionary bridge between land and aquatic plants, and produces typical aquatic leaves if grown under water. In this report, we show that the heterophyllic leaf development of this plant is mainly determined by ABA and ethylene signalings which regulate leaf polarity genes. In aquatic environments, ethylene level is increased and the ethylene signaling overactivates the expression of abaxial genes, *RtKANs*, which antagonistically suppresses adaxial genes, *RtHD-ZIPIIIs*. Such overexpression of abaxial genes is most likely the molecular mechanism behind the cylindrical shape of aquatic leaves. In contrast, in terrestrial environments, ABA level is increased and ABA signaling activates the expression of adaxial genes, *RtHD-ZIPIIIs*, which establishes adaxial-abaxial polarity and causes broad leaf development (Fig 4E).

ABA is a well-known stress hormone in plants; it is accumulated by various abiotic and biotic stresses and confers resistance against them [38, 39]. Since flooding is also a stress to land plants, it is plausible that myriad of land plants show increased levels of ABA after submergence [40, 41]. Such ABA accumulation seems to be evolutionarily adaptive to land plants because it renders the plants to adopt ‘stunt strategy’; enduring the flooding period by inducing growth retardation which restricts energy consumption [42]. However, submergence-tolerant species such as deepwater rice and *Rumex palustris* have evolved differently. They show the opposite response to submergence in which ABA contents decreased [6, 43]. It indicates that reducing ABA level is adaptive to aquatic environments in some plant species. Consistent with this, ABA contents and the expression levels of *RtAAO*, a gene involved in a critical step of ABA biosynthesis, are decreased in *R*. *trichophyllus* in aquatic condition. It is likely that the suppression of ABA biosynthesis under water is widely occurred among submergence-tolerant plants and *R*. *trichophyllus* has also adopted similar evo-devo mechanism during evolution for aquatic adaptation.

Increase of ethylene level by flooding is also observed among various plant taxa, which is achieved by the enhancement of biosynthesis or local entrapment by submergence [7]. In addition, in wide range of plants from moss to Arabidopsis, the treatment of exogenous ethylene mimics submerged growth [44, 45]. Therefore, it is likely that the activation of ethylene signaling is a widely conserved response to submergence in plants. Consistently, ethylene biosynthesis and signaling are increased in *R*. *trichophyllus* by submergence. Interestingly, the antagonistic interaction between ABA and ethylene found in heterophylly of *R*. *trichophyllus* is also observed in many developmental processes in plants. For example, hyponastic growth of leaf in submerged *R*. *palustris* is regulated by the antagonistic interaction of ABA and ethylene [8]. Therefore, heterophylly of *R*. *trichophyllus* seems to be evolved from the common mechanism of ABA/ethylene interaction observed in other land plants.

Based on the roles of leaf polarity genes known in *A*. *thaliana*, differential expressions of leaf polarity genes in *R*. *trichophyllus* seem to lead to the three developmental changes which are required for adaptation to aquatic environments. That is, cylindrical shape leaves and reduced numbers of stomata seem to be caused by overactivation of the abaxial genes, *RtKANs*, as ectopic expression of *KAN1* or *KAN2* in Arabidopsis throughout the leaf primordia results in abaxialized radial organs, with a concomitant loss of *HD-ZIPIII* expression [14, 16]. Subsequently, the loss of *RtHD-ZIPIIIs* seems to cause reduced number of vessel elements, similar to loss-of-function of *HD-ZIPIII* genes in Arabidopsis showing reduced xylem [14]. Recent reports showed that a KNOX-GA module is critical for the heterophyllic development of *Rorippa aquatica* [36], which is a different mechanism than that we have found in *R*. *trichophyllus*. KNOX-GA module seems not to be a main mechanism for the heterophylly in *R*. *trichophyllus*. First, morphological pattern of heterophylly is not similar in the two species (S8A Fig). Heterophylly of *R*. *aquatica* is achieved by deep serration of leaves, thus it changes simple leaves to dissected compound leaves. In contrast, in case of *R*. *trichophyllus*, leaf complexity is increased in both terrestrial and aquatic leaves during growth and leaf branching pattern is not significantly different between terrestrial and aquatic leaves (S8A Fig). Instead, heterophylly of *R. trichophyllus* is achieved by leaf elongation and radialization rather than leaf serration. Second, GA has little effect on the heterophylly of *R*. *trichophyllus* although it is a main participant of the heterophylly in *R*. *aquatica* (Fig 3D and 3E). Thus, the heterophyllic developments in *R*. *tricophyllus* and *R*. *aquatica* have adopted different mechanisms, indicating that convergent evolution has occurred.

In spite of such differences in overall architecture, there is some convergent point between *R*. *trichophyllus* and *R*. *aquatica*. Like *R*. *aquatica*, aquatic leaves were generated by cold environment in *R*. *trichophyllus* (Fig 7). It is still unclear why amphibious plants induce aquatic leaf development in response to cold stress. Wells and Pigliucci (2000) proposed an “anticipatory plasticity hypothesis” in which plants can show similar phenotypic plasticity in response to different external cues coming together in nature. For example, submergence into water in nature causes combination of changes in diverse environmental cues, e.g. humidity, temperature, changes in light quality, etc. Thus, cold and hypoxia-induced molecular changes in the expression of leaf polarity genes in *R*. *trichophyllus* seem to be supportive to our hypothesis that changes in leaf polarity drive the evolution of amphibious adaptation in *R*. *trichophyllus*. The more direct evidence will be obtained through the analyses of mutants and transgenic plants which show defects in the heterophyllic development. In that sense, the recent report suggesting *Hygrophilia difformis* as a model plant to study heterophylly of amphibious plant is interesting [46]. *H*. *difformis* is easy to grow and propagated vegetatively well and above all, it can be easily transformed by *Agrobacterium tumefaciens*. However, the molecular basis of heterophylly of *H*. *difformis* is similar to that of *R*. *aquatica* but is different with that of *R*. *trichophyllus*. For example, in *H*. *difformis*, GA is a major regulator determining heterophylly and aquatic leaf development is achieved by deep serration of leaves, thus changing simple leaves to dissected compound leaves. Such characteristics are very similar with those of *R*. *aquatic* but dissimilar to those of *R*. *trichophyllus*. Therefore, *H*. *difformis* as a model plant for amphibious plants is limiting.

From comparative studies using two land plants, *A*. *thaliana* and *R*. *sceleratus*, and one amphibious plant, *R*. *trichophyllus*, we found that at least two molecular changes have occurred in *R*. *trichophyllus* during evolutionary adaptation to aquatic environments; the suppression of ABA biosynthesis and abaxialization of leaf development. Since ABA signaling components and the regulatory mechanism of HD-ZIPIII transcription factors seem to have evolved during land colonization by plants [24, 47], such molecular changes observed in *R*. *trichophyllus* are suggestive of evolutionary trend rearranging pre-existing gene networks instead of generating novel one [48]. Since many aquatic plants share similar morphological traits observed in submerged *R*. *trichophyllus*, further analysis of this plant will provide deep insight into the understanding of convergent evolution occurred in aquatic plants.

## Methods

### Plant materials and growth conditions

Seeds of *Ranunculus trichophyllus* var. *kadzusensis* were collected from its native habitat at Ganghwa Island, South Korea. Seeds of *Ranunculus sceleratus*, collected from Namyangju City, were donated from the Korea National Arboretum. Seeds were sterilized with 70% ethanol and with 1% NaOCl and 0.5% Tween-20 solution. Seeds were sown on half-strength Murashige-Skoog (MS) medium containing 50 μM carbenicilin, 75 μM cefotaxim, and 1% agar. Seeds of *R*. *trichophyllus* and *R*. *sceleratus* were germinated on solid MS media for 1 week, at that time root radicles were just protruded. Then germinated seedlings are transferred to aerial or aquatic/submerged environments. The true leaves produced at 7 days after transference were used for morphological analysis and RNA expression analysis. For transcriptome analysis, the plants 10 days after transference were used for RNA expression. In case of Arabidopsis, 4 days-old seedlings after germination were transplanted, then submerged into water for 2 weeks. For *Arabidopsis thaliana*, Col-0 seeds were used. The growth room was maintained at 22°C, 60 ± 10% relative humidity in long day photoperiod (16h light/8h dark).

### RNA sequencing analyses

cDNA libraries were obtained using 1 μg of total RNA extracted from whole plant tissues of *R*. *trichophyllus*. 100 base pair paired-end libraries were sequenced by Illumina HiSeqTM 2000. The libraries were quantified according to the qPCR Quantification Protocol Guide and qualified using an Agilent Technologies 2100 Bioanalyzer. RNA-seq reads were de novo assembled and mapped using Trinity and TopHat programs and the relative transcript levels were calculated by FPKM (Fragments Per Kilobase of exon per Million fragments mapped) using Cufflinks software. Excluded transcripts were filtered with 1 FPKM value and transformed to logarithm scale. They are normalized by quantile normalization method. Transcripts were assigned a putative function, then gene ontology analysis was performed by using DAVID tool (http://david.abcc.ncifcrf.gov/).

### Hormone treatments

One-week-old seedlings of *R*. *trichophyllus* were used. The concentrations of hormones used were 10 μM NAA (1-naphthaleneacetic acid, Duchefa, N0903), 50 μM ACC (1-aminocyclopropane-1-carboxylic acid, Sigma Aldrich, A3903), 1 μM EBL (epi-brassinolide, Sigma Aldrich, E1641), 50 μM bikinin (Sigma Aldrich, SML0094) and 10 μM gibberellin (GA, bioWorld, 714248), respectively. For submerged treatment, abscisic acid (ABA, Sigma Aldrich, A0149), silver nitrate (AgNO_3_, Sigma Aldrich, S8157), and paclobutrazol (PBZ, Sigma Aldrich, 46046) were added into the aquatic media. The concentrations used were 1 μM ABA, 10 μM AgNO_3_, and 10 μM PBZ, respectively. After 10 days of growth, the first true leaves from the seedlings were analyzed.

### Stomata and vessel elements

For whole mount clearing, first true leaves were soaked in clearing solution (2.5 g chloral hydrate; 0.3 ml 100% glycerol; 0.7 ml distilled water), then incubated for 3 h at 55°C. The epidermis and xylem elements were observed using an Axio Imager A1 microscope (Carl Zeiss) under DIC optics. Images were captured using an AxioCam HRc camera (Carl Zeiss).

### Hormonal contents

Seedlings (using about 20 individual seedlings) were grown in MS with or without 150 ml distilled water. Using 3 ml disposable syringe, we harvested capped air in headspace containing ethylene, then sealed by parafilm. Using Hamilton syringe, 100 μl gas was extracted from sealed air, then feeding to gas chromatography with flame ionization detector (Agilent 7890B GC). We used HP-5 column (#19091J-413, Agilent). Ethylene production was normalized by seedling weight. For measuring ABA contents, we used ABA ELISA kit (CSB-E09159Pl). Intensity of 450 nm fluorescence was determined by using Plate reader-Powerwave X (Bio-Tek). ABA production was normalized by sample weight. The measurements were performed from three biological replicates and two technical replicates each.

### Sequencing of orthologous genes

Candidate genes were selected using information of *A*. *thaliana* and the TAIR database (www.arabidopsis.org). The Arabidopsis sequences were used to search for orthologous genes from *Aquilegia coerulea*, for which the genome database (http://www.phytozome.net/search.php?method=Org_Acoerulea) is available. *Aquilegia coerulea* is the closest relative to the Ranunculus genus among plants that have an available sequence database.

### Gene expression studies using real-time PCR

For real time-qPCR, total RNA was isolated from leaves using TRI reagent (Sigma Aldrich, T9424) and RNeasy Plant Mini Kit (Qiagen, 74904). cDNA was generated using 4 μg of total RNA, 5 unit of reverse transcriptase (Fermentas, EP0442), 4 μl of 2.5 mM dNTP, 2 μl of 50 mM oligo(dT), and ddH2O to 40 μl. For real time qPCR, 0.3 μl of synthesized cDNA was mixed with 2 μl of 5 μM primers and 10 μl of SYBR Green qPCR Master Mix (Bio-Rad), and ddH2O to 20 μl. Real-time-qPCR analysis was performed by CFX96 Real-Time PCR system (Bio-Rad). The relative transcript levels were calculated according to the ΔΔCt method. [49]

### Protoplasts transfection assay

Leaflets of *R*. *trichophyllus* which was grown on short days (8 h light/16 h dark, 22°C) were used for the isolation and transfection of protoplasts. The method of transfection was based on previously described.[50] For transfection, we used 10% PEG final concentration. After 1 day of incubation, the protoplasts were harvested for real time-qPCR and luciferase activity assays. For determining promoter activity, we used luciferase assay system (Promega, E1500) and microplate luminometer (Berthold).

### Phylogenetic analyses

Multiple alignment of amino acid sequences was performed using the ClustalX2.1 program (http://www.clustal.org/download/current/), which generates aligned phy format files. These aligned files were passed through the PHYLIP program (version 3.69) for phylogenetic analyses (http://evolution.genetics.washington.edu/phylip.html). In the PHYLIP software, SEQBOOT, PROTDIST, NEIGHBOR, and CONSENSE programs were run sequentially to generate draft unrooted phylogenetic trees and to obtain bootstrap values. The phylogenetic tree was drawn using the TreeView program (http://taxonomy.zoology.gla.ac.uk/rod/treeview.html).

### Whole mount *in situ* hybridization

All *in situ* hybridization experiments were performed as described previously.[51] For signal detection using NBT/BCIP (Roche, 11681451001), 100 ng of DIG-labelled RNA probes per mL of ALP buffer was used for hybridization. The images were obtained by light microscopy.

For fluorescence detection using HNPP (2-hydroxy-3-naphtoic acid-2'-phenylanilide phosphate, Roche, 1758888001), leaves were hybridized with DIG-labelled RNA probes, then stained with a mixture of 10 μl HNPP and 0.25 mg Fast Red TR solution per mL in ALP buffer containing 2 mM levamisole for 30 min at room temperature. Leaves were washed in distilled water for 10 min and incubated with 0.2 μg per mL DAPI (4,6-diamidino-2-phenylindole) for 10 min at RT for nuclear counter-staining. Fluorescence was detected by confocal laser scanning microscopy (LSM700, Carl Zeiss).

### Statistical analysis

Statistical analyses were performed using an unpaired Student’s *t*-test. For multiple comparisons, we used a one-way ANOVA and post-hoc test. We considered *P* < 0.05 as statistically significant. All statistical analyses were performed using the statistical package R.[52]

## Supporting information

S1 Fig Effects of long-term submergence on the three plants, *R*. *trichophyllus*, *R*. *sceleratus*, and Arabidopsis. (A) Cartoon depicting habitats of *R*. *trichophyllus*, *R*. *sceleratus*, and *A*. *thaliana*. (B) Effects of long-term submergence on the plant growth. For *R*. *trichophyllus* and *R*. *sceleratus*, 1 week-old seedlings after germination on the MS media were transferred to aquatic condition for 3 weeks. For Arabidopsis, 4 day-old seedlings after germination were transferred to aquatic condition for 2 weeks.
(TIF)

S2 Fig **Comparison of gene expressions between terrestrial vs aquatic/submerged plants of *R*. *trichophyllus* (A) and *R*. *sceleratus* (B).** Differential expressions of genes affiliated to GO terms for defense, wax, stomata, and vasculature were compared. The ortholog of *RtPDF1*.*4* for *R*. *sceleratus* could not be cloned. For submergence, two weeks old plants grown on solid MS media were submerged into water for 5 days for RNA extraction.
(TIF)

S3 Fig Effects of auxin and brassinosteroid on the leaf index of terrestrial leaves of *R*. *trichophyllus*. (A) Effect of auxin agonist, NAA, on the leaf index. (B) Effects of brassinosteroid inhibitor, EBL, and an agonist, bikinin, on the leaf index. The data are presented as means ± SD from three biological replicates.
(TIF)

S4 Fig Effects of ethylene and ABA on the leaf morphologies of *R*. *sceleratus*. (A) Land-grown plants treated with (+: lower panel) or without (control: upper panel) ethylene precursor ACC. 1 week-old seedlings were treated with ethylene, then analyzed after 7 days. (B) Submerged plants treated with (+: lower panel) or without (control: upper panel) ABA. 1 week-old seedlings were submerged into ABA-containing water, then analyzed after 10 days. Images from left to right, seedling morphologies, microscopic structure of cell shapes, vessel elements in petiole. Black arrowhead denotes stomata, white arrowhead denotes vessel elements.
(TIF)

S5 Fig Phylogenetic trees of orthologs of *KAN* and *HD-ZIPIII* gene families. (A and B) The families of *KAN* (A) and *HD-ZIPIII* (B) genes from *R*. *trichophyllus* and *R*. *sceleratus* were aligned with those of *A*. *thaliana* based on amino acid sequences. *AtPSR1* and *AtML1* were used as out-groups. Bootstrap values are denoted beside branch nodes. Only values greater than 75 are presented.
(C) Overexpression lines of *RtKAN* and *RtHD-ZIPIII* genes can provoke abnormal leaf formation. 2 weeks-old seedlings were fictured.
(TIF)

S6 Fig Whole mount *in situ* hybridization of *RtKANa* and *HD-ZIPIIIa* by HNPP staining. DAPI is in blue and HNPP signal for *RtKANa* or *RtHD-ZIPIIIa* is in red. The right panels show the merged fluorescence of DAPI and HNPP. **A-C.** Hybridization signals for *RtKANa* detected in terrestrial leaves (A), aquatic leaves (B), and terrestrial leaves treated with ethylene (C). **D-F.** Hybridization signals for *RtHD-ZIPIIIa* detected in terrestrial leaves (D), aquatic leaves (E), and aquatic leaves treated with ABA (F). Ab, abaxial side; Ad, adaxial side.
(TIF)

S7 Fig Expressions of leaf polarity genes from *R*. *sceleratus* and *A*. *thaliana* are not affected by submergence. Comparison of transcript levels of *KANs* and *HD-ZIPIIIs* from *R*. *sceleratus* (A) and *A*. *thaliana* (B) before and after submergence. For submergence, two weeks old plants grown on solid MS media were submerged into water for 5 days for RNA extraction.
(TIF)

S8 Fig Leaf architecture is similar between terrestrial and aquatic leaves. (A) Terrestrial (left panel) and aquatic leaves (right panel) from 1^st^ true leaves. Upper row is 1^st^ to 3^rd^ leaves and lower row is 4^th^, 5^th^, and 6^th^ leaves.
(B) 1^st^ leaves of terrestrial leaves (left panels) and aquatic leaves (right panels).
(TIF)
