# Supplementary figures and images for: A molecular basis behind heterophylly in an amphibious plant, Ranunculus trichophyllus
Source: PLoS Genet. 2018 Feb 15;14(2):e1007208. doi: 10.1371/journal.pgen.1007208 (PMC5831646; doi:10.1371/journal.pgen.1007208)

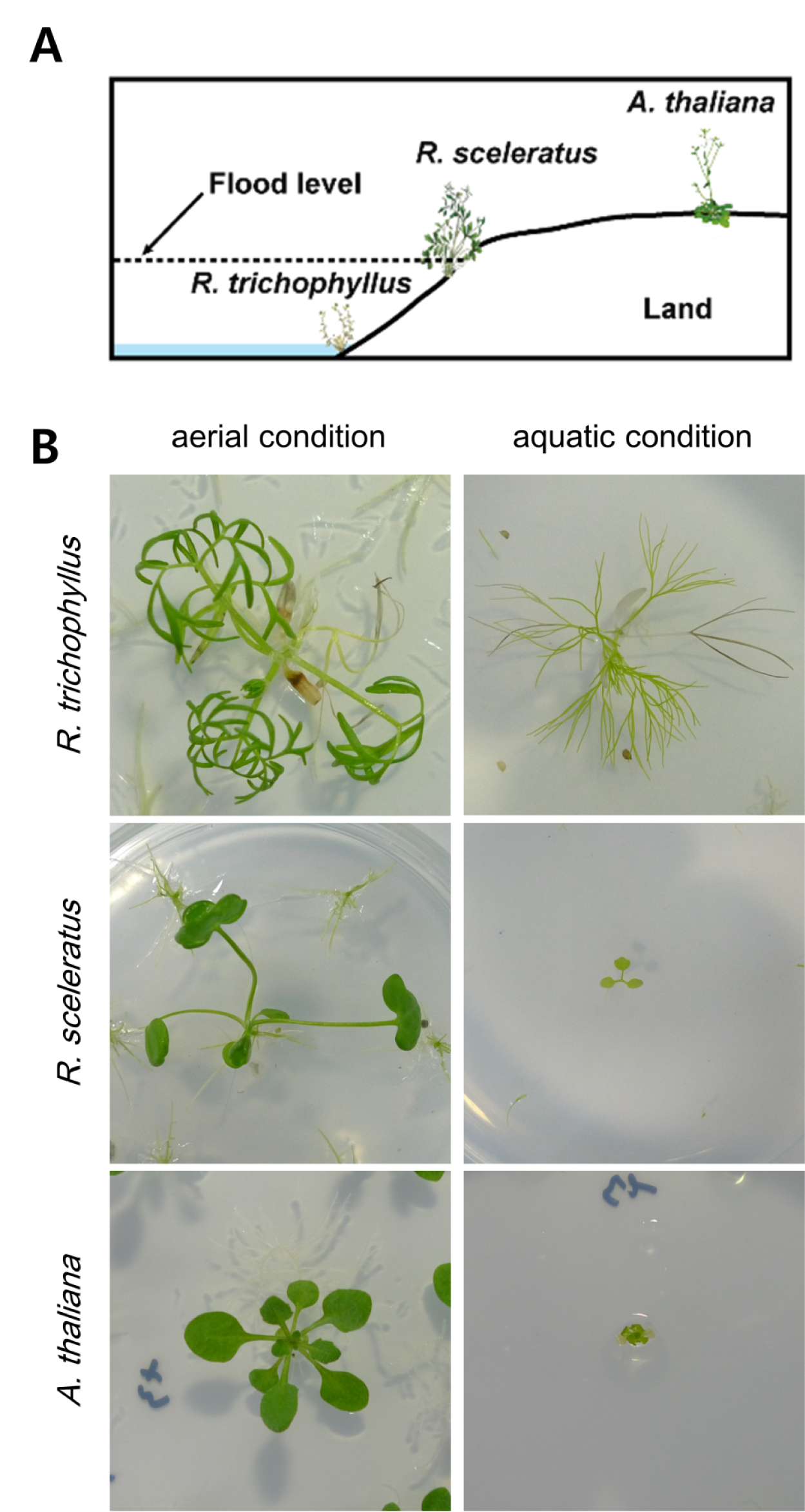

Supplement: S1 Fig — (A) Cartoon depicting habitats of R. trichophyllus, R. sceleratus, and A. thaliana. (B) Effects of long-term submergence on the plant growth. For R. trichophyllus and R. sceleratus, 1 week-old seedlings after germination on the MS media were transferred to aquatic condition for 3 weeks. For Arabidopsis, 4 day-old seedlings after germination were transferred to aquatic condition for 2 weeks. (TIF) [file pgen.1007208.s001.tif]

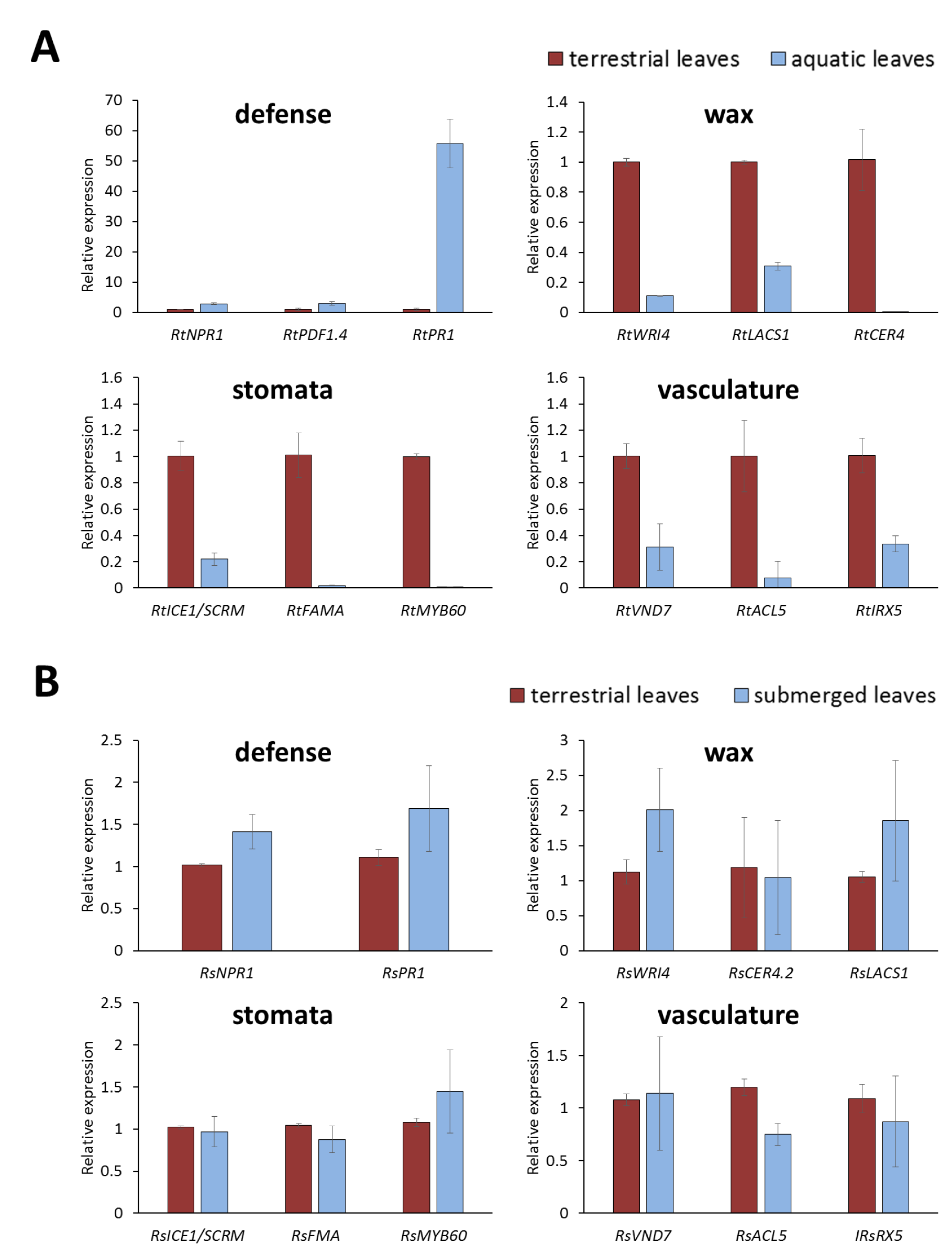

Supplement: S2 Fig — Comparison of gene expressions between terrestrial vs aquatic/submerged plants of R. trichophyllus (A) and R. sceleratus (B). Differential expressions of genes affiliated to GO terms for defense, wax, stomata, and vasculature were compared. The ortholog of RtPDF1.4 for R. sceleratus could not be cloned. For submergence, two weeks old plants grown on solid MS media were submerged into water for 5 days for RNA extraction. (TIF) [file pgen.1007208.s002.tif]

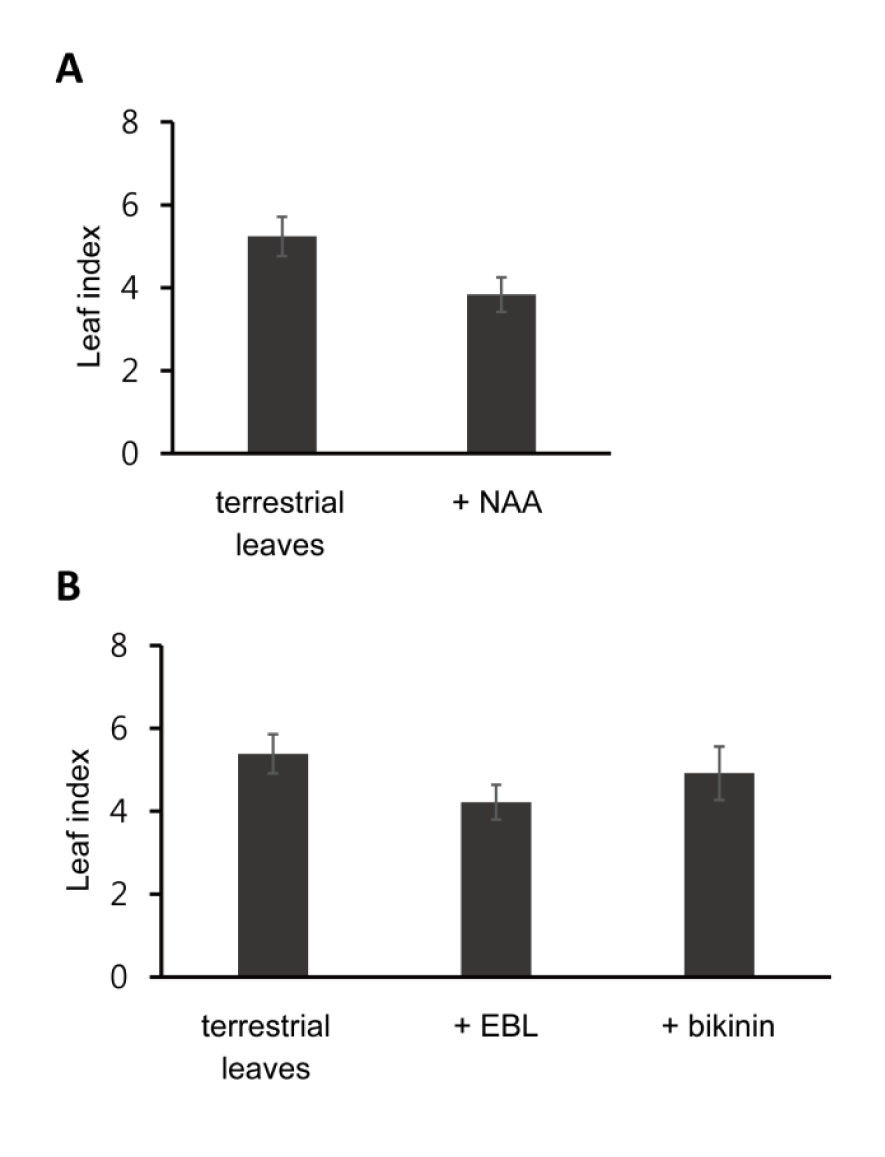

Supplement: S3 Fig — (A) Effect of auxin agonist, NAA, on the leaf index. (B) Effects of brassinosteroid inhibitor, EBL, and an agonist, bikinin, on the leaf index. The data are presented as means ± SD from three biological replicates. (TIF) [file pgen.1007208.s003.tif]

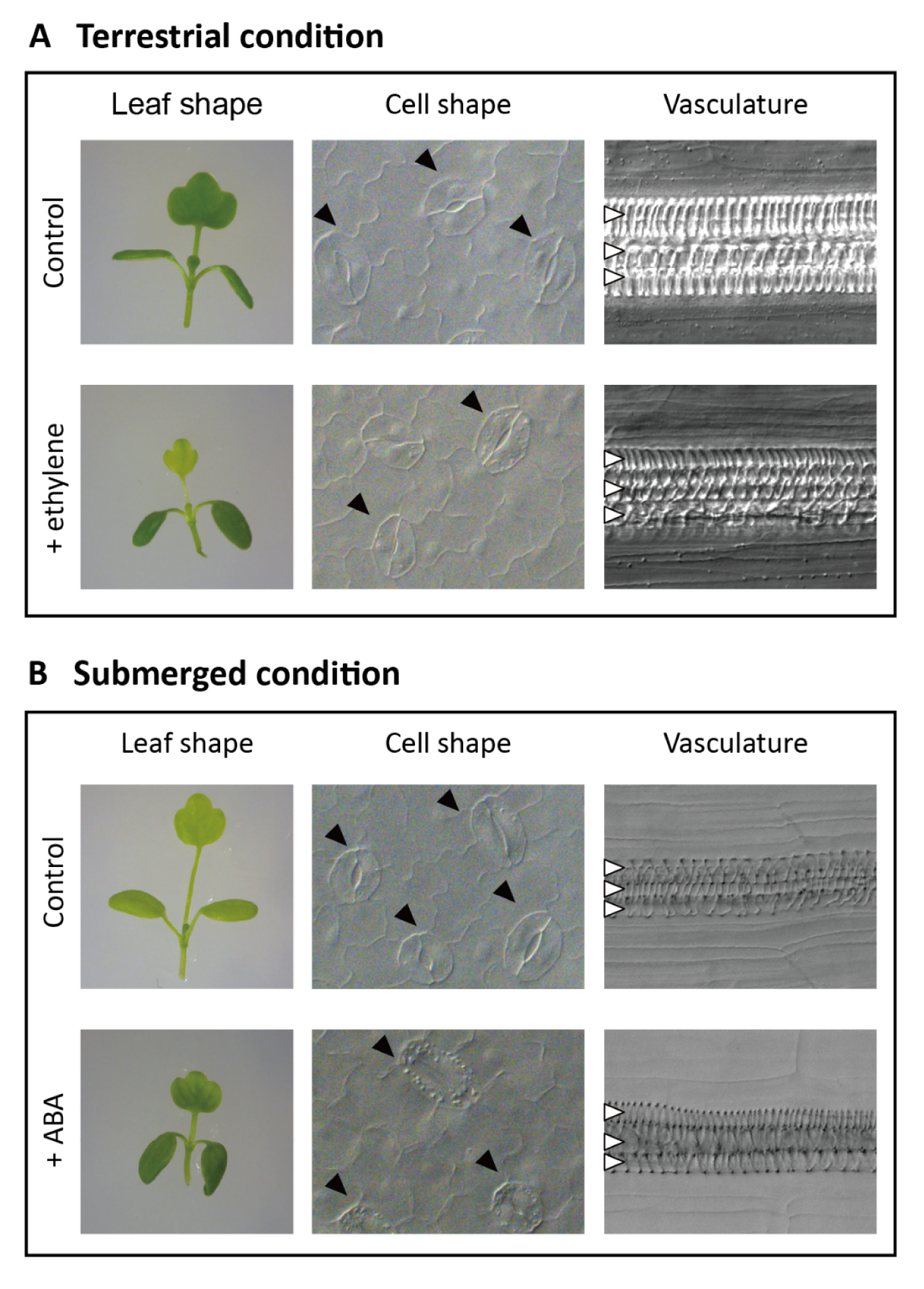

Supplement: S4 Fig — (A) Land-grown plants treated with (+: lower panel) or without (control: upper panel) ethylene precursor ACC. 1 week-old seedlings were treated with ethylene, then analyzed after 7 days. (B) Submerged plants treated with (+: lower panel) or without (control: upper panel) ABA. 1 week-old seedlings were submerged into ABA-containing water, then analyzed after 10 days. Images from left to right, seedling morphologies, microscopic structure of cell shapes, vessel elements in petiole. Black arrowhead denotes stomata, white arrowhead denotes vessel elements. (TIF) [file pgen.1007208.s004.tif]

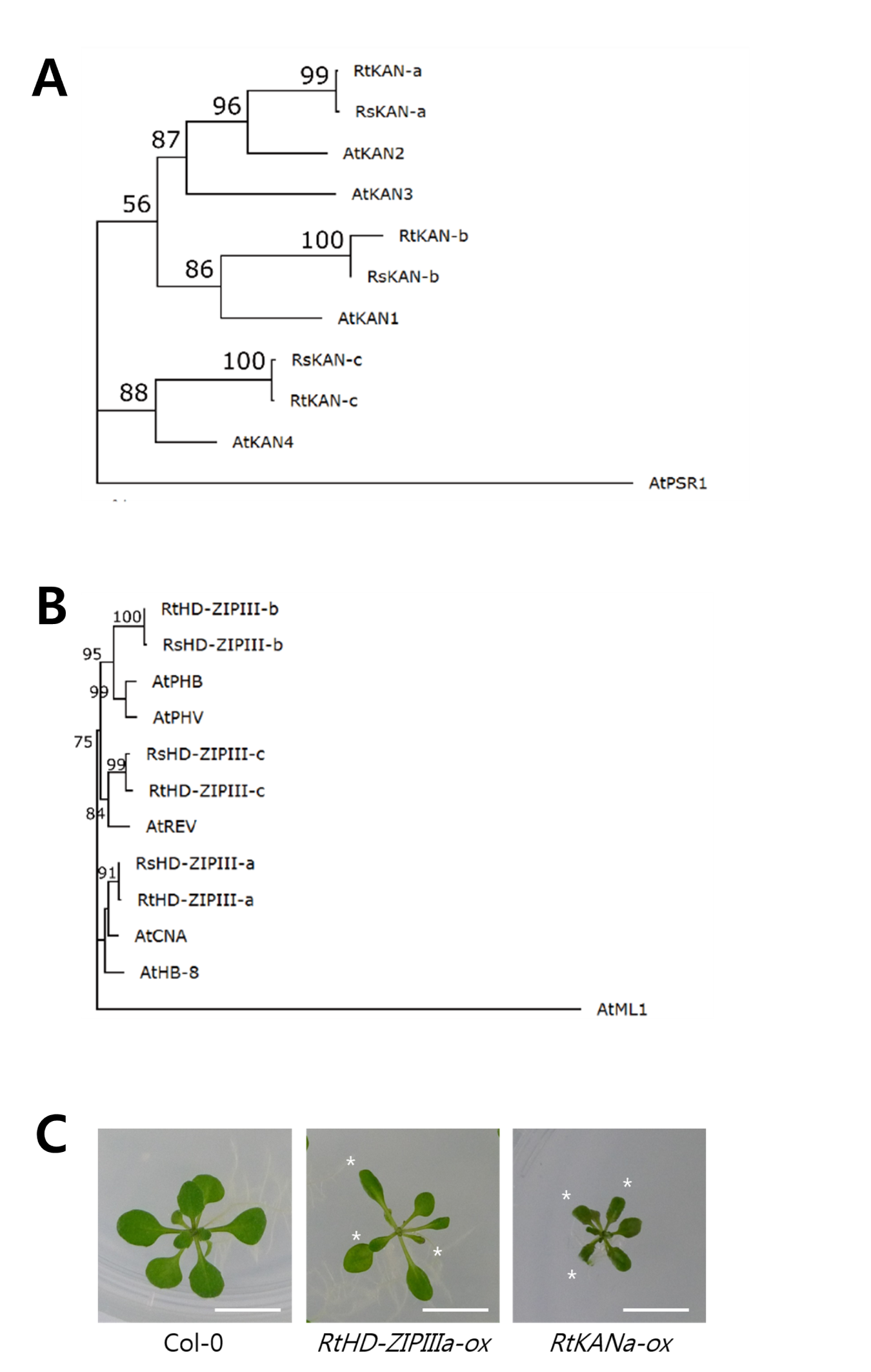

Supplement: S5 Fig — (A and B) The families of KAN (A) and HD-ZIPIII (B) genes from R. trichophyllus and R. sceleratus were aligned with those of A. thaliana based on amino acid sequences. AtPSR1 and AtML1 were used as out-groups. Bootstrap values are denoted beside branch nodes. Only values greater than 75 are presented. (C) Overexpression lines of RtKAN and RtHD-ZIPIII genes can provoke abnormal leaf formation. 2 weeks-old seedlings were fictured. (TIF) [file pgen.1007208.s005.tif]

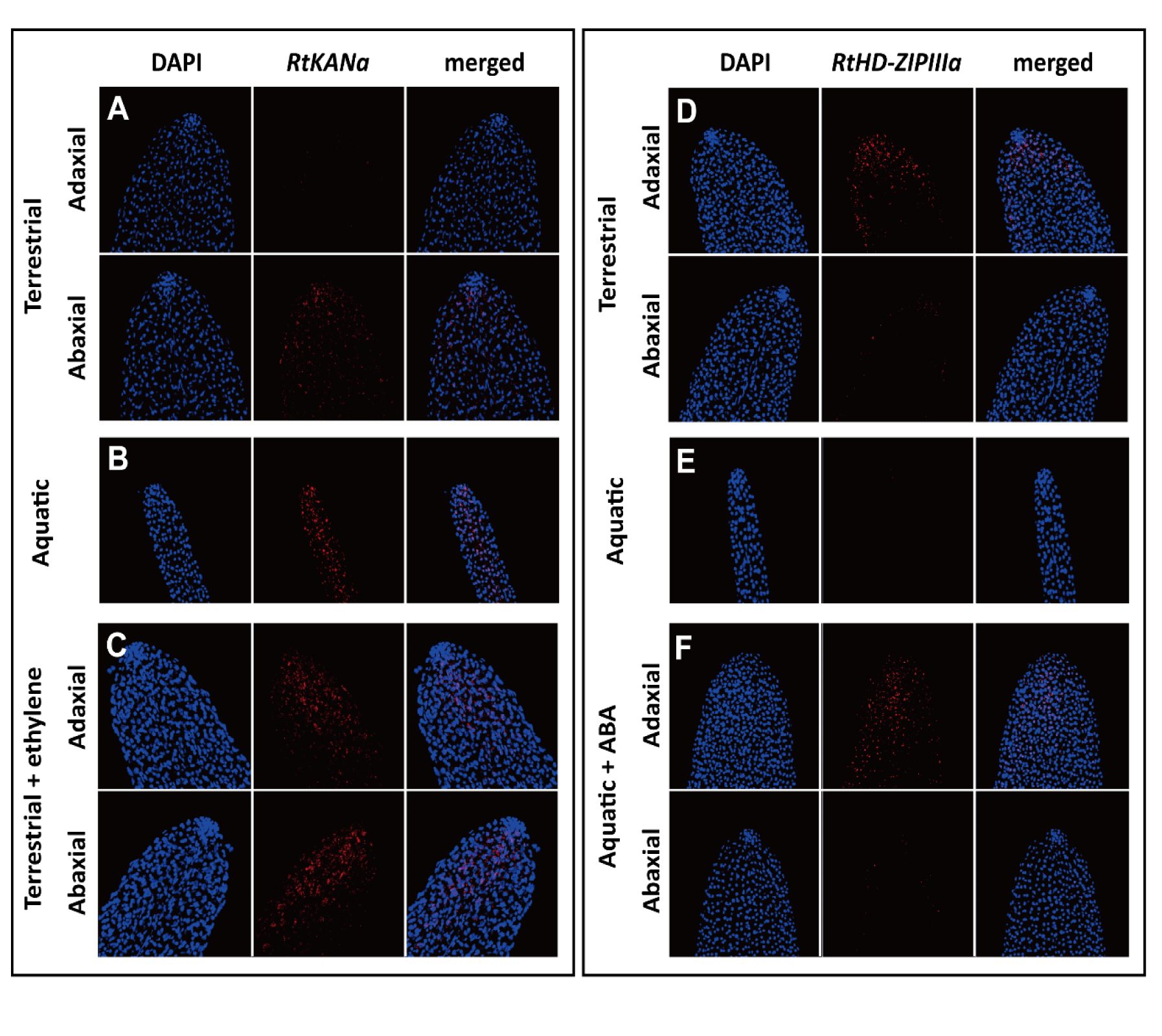

Supplement: S6 Fig — DAPI is in blue and HNPP signal for RtKANa or RtHD-ZIPIIIa is in red. The right panels show the merged fluorescence of DAPI and HNPP. A-C. Hybridization signals for RtKANa detected in terrestrial leaves (A), aquatic leaves (B), and terrestrial leaves treated with ethylene (C). D-F. Hybridization signals for RtHD-ZIPIIIa detected in terrestrial leaves (D), aquatic leaves (E), and aquatic leaves treated with ABA (F). Ab, abaxial side; Ad, adaxial side. (TIF) [file pgen.1007208.s006.tif]

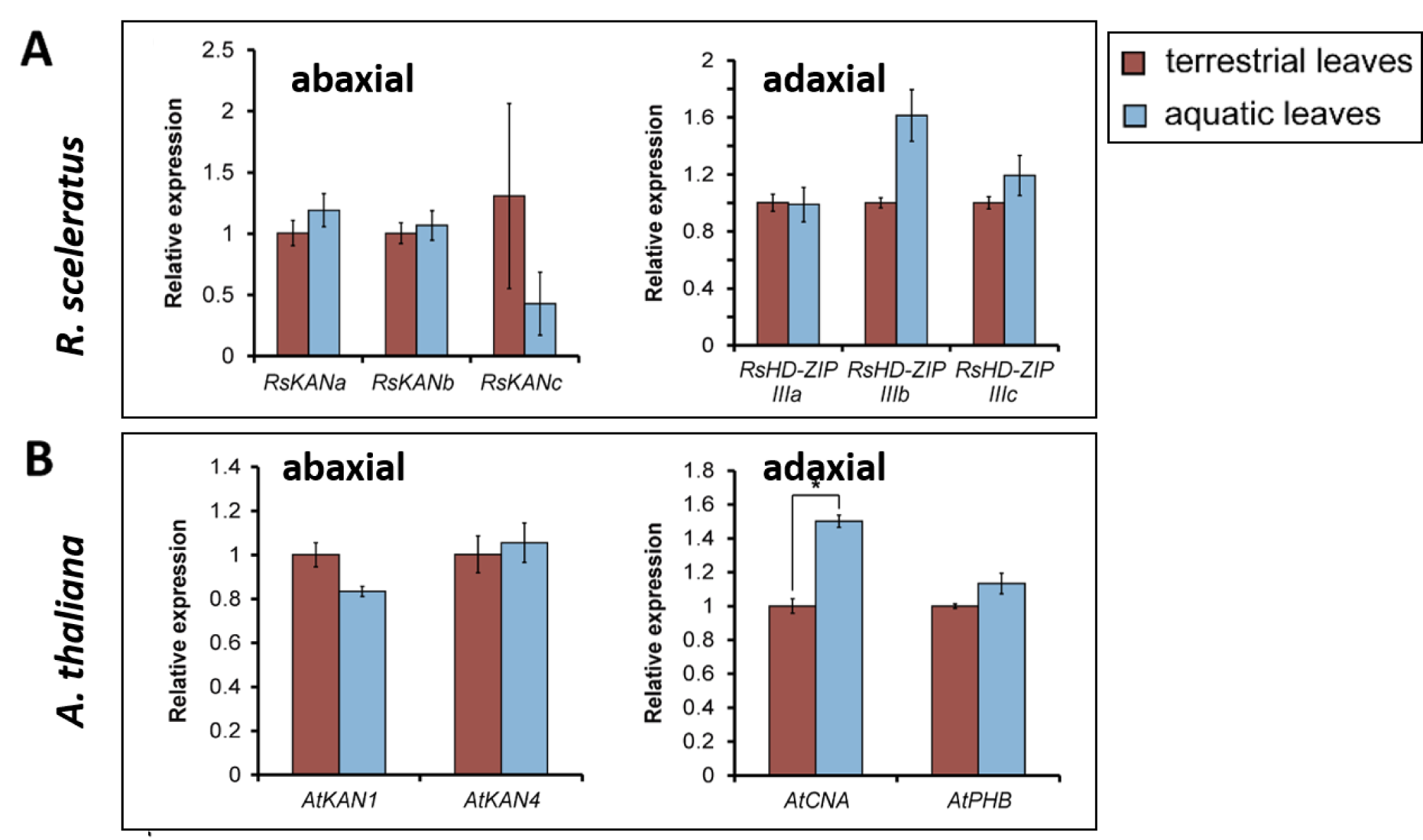

Supplement: S7 Fig — Comparison of transcript levels of KANs and HD-ZIPIIIs from R. sceleratus (A) and A. thaliana (B) before and after submergence. For submergence, two weeks old plants grown on solid MS media were submerged into water for 5 days for RNA extraction. (TIF) [file pgen.1007208.s007.tif]

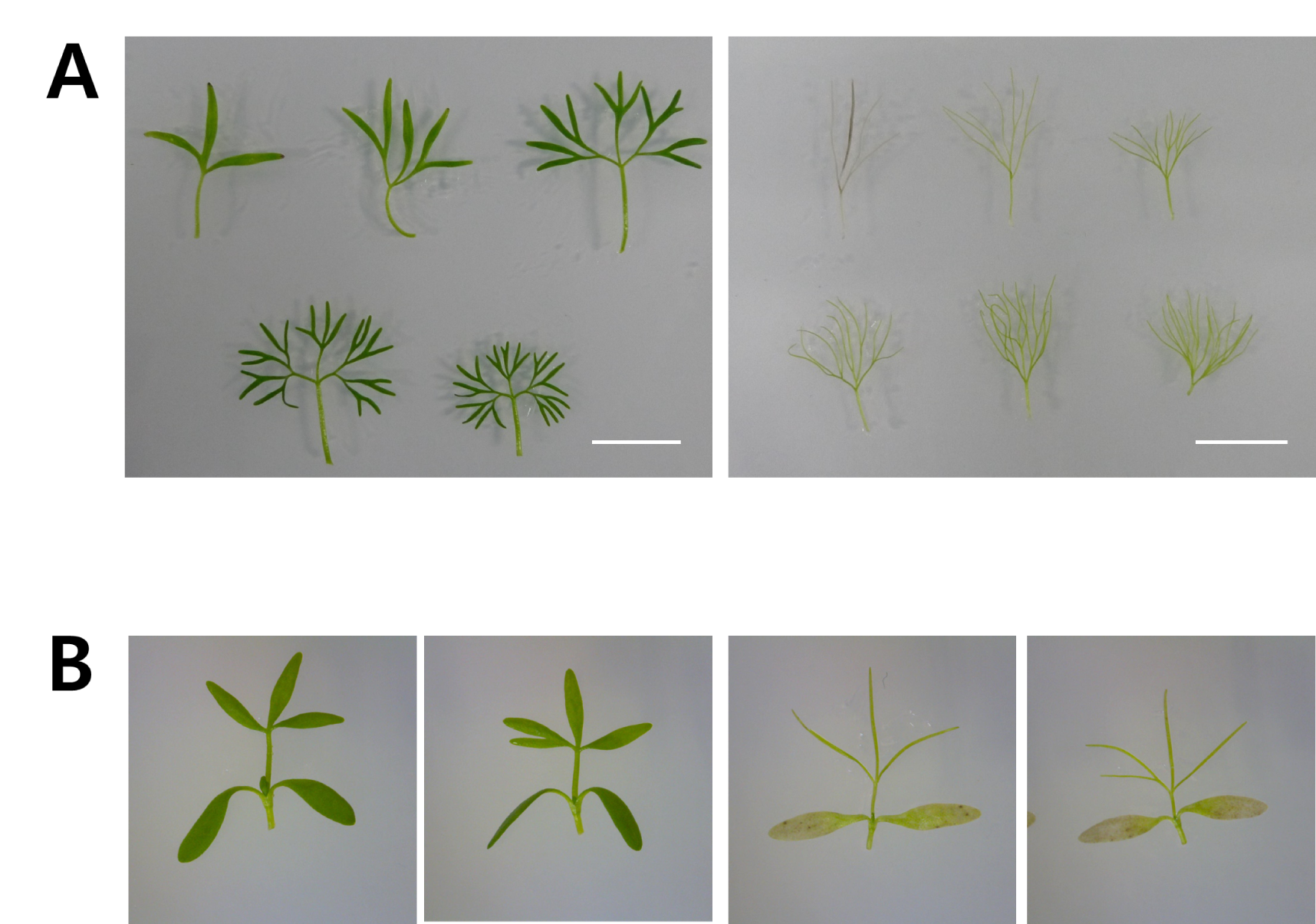

Supplement: S8 Fig — (A) Terrestrial (left panel) and aquatic leaves (right panel) from 1st true leaves. Upper row is 1st to 3rd leaves and lower row is 4th, 5th, and 6th leaves. (B) 1st leaves of terrestrial leaves (left panels) and aquatic leaves (right panels). (TIF) [file pgen.1007208.s008.tif]
